# Burst firing in Output‐Defined Parallel Habenula Circuit Underlies the Antidepressant Effects of Bright Light Treatment

**DOI:** 10.1002/advs.202401059

**Published:** 2024-06-11

**Authors:** Xianwei Liu, Han Li, Ruijia Ma, Xiaohan Tong, Jijin Wu, Xiaodan Huang, Kwok‐Fai So, Qian Tao, Lu Huang, Song Lin, Chaoran Ren

**Affiliations:** ^1^ Department of Neurology and Stroke Center First Affiliated Hospital of Jinan University Key Laboratory of CNS Regeneration (Ministry of Education) Guangdong Key Laboratory of Non‐human Primate Research GHM Institute of CNS Regeneration Jinan University Guangzhou 510632 China; ^2^ Physiology Department Key Laboratory of Viral Pathogenesis & Infection Prevention and Control, School of Medicine Jinan University Guangzhou 510632 China; ^3^ Co‐innovation Center of Neuroregeneration Nantong University Nantong 226001 China; ^4^ Neuroscience and Neurorehabilitation Institute University of Health and Rehabilitation Sciences Qingdao 266113 China; ^5^ Department of Rehabilitation Medicine First Affiliated Hospital of Jinan University Psychology Department School of Medicine Jinan University Guangzhou 510632 China

**Keywords:** bright light treatment, burst firing, depression, lateral habenula, neural circuits

## Abstract

Research highlights the significance of increased bursting in lateral habenula (LHb) neurons in depression and as a focal point for bright light treatment (BLT). However, the precise spike patterns of LHb neurons projecting to different brain regions during depression, their roles in depression development, and BLT's therapeutic action remain elusive. Here, LHb neurons are found projecting to the dorsal raphe nucleus (DRN), ventral tegmental area (VTA), and median raphe nucleus (MnR) exhibit increased bursting following aversive stimuli exposure, correlating with distinct depressive symptoms. Enhanced bursting in DRN‐projecting LHb neurons is pivotal for anhedonia and anxiety, while concurrent bursting in LHb neurons projecting to the DRN, VTA, and MnR is essential for despair. Remarkably, reducing bursting in distinct LHb neuron subpopulations underlies the therapeutic effects of BLT on specific depressive behaviors. These findings provide valuable insights into the mechanisms of depression and the antidepressant action of BLT.

## Graphical abstract

This study demonstrates that LHb neurons projecting to the DRN, VTA, and MnR exhibit increased bursting following aversive stimuli exposure, correlating with distinct depressive symptoms. Furthermore, decreased bursting in specific LHb neuron subpopulations underlies the therapeutic effects of bright light treatment (BLT) on specific depressive‐like behaviors. These findings deepen insights into depression mechanisms and BLT's antidepressant effects.

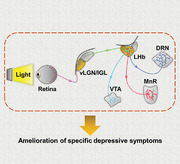

## 1 Introduction

Depression, a widespread mental health disorder, affects over 120 million individuals worldwide, leading to a significant societal burden.^[^ ^1^ ^]^ This condition is characterized by symptoms such as anhedonia and despair and often coexists with anxiety disorders.^[^ ^2^ ^]^ Despite significant progress in comprehending depression, the specific pathological mechanisms underlying these conditions remain incompletely understood, hampering the development of innovative therapeutic strategies.

The lateral habenula (LHb), a component of the epithalamus, plays a crucial role in encoding stress signals.^[^ ^3^ ^]^ It directly projects to downstream aminergic reward centers, including the dorsal raphe nucleus (DRN), ventral tegmental area (VTA), and median raphe nucleus (MnR).^[^ ^3^, ^4^ ^]^ Excessive activity in the LHb has been associated with depressive‐like symptoms in both human patients and animal models.^[^ ^3^, ^5^ ^]^ Recent rodent studies have demonstrated a correlation between increased burst firing in LHb neurons and depressive‐like behaviors.^[^ ^6^ ^]^ However, the exact alterations in the spike patterns of LHb neurons projecting to these aminergic centers during depression and their role in the development of depressive pathology have not yet been fully investigated.

Light signals transmitted through the retina have a significant impact on mood‐related behaviors.^[^ ^7^ ^]^ Our recent study demonstrated that bright light treatment (BLT) effectively reduces burst firing in the LHb and alleviates depressive‐like behaviors via the retina→ventral lateral geniculate nucleus and intergeniculate leaflet (vLGN/IGL)→LHb pathway.^[^ ^3a^ ^]^ However, the precise downstream targets of LHb neurons and the underlying mechanisms responsible for mediating the antidepressant effects of BLT have not yet been identified.

In this study, we identified three distinct subpopulations of LHb neurons, characterized by their projections to the DRN (LHb^→DRN^), VTA (LHb^→VTA^), and MnR (LHb^→MnR^), which exhibited increased bursting in mice that received long‐term exposure to aversive stimuli (AS). We found that bursting in these LHb neuronal subpopulations mediates distinct depressive‐like symptoms. Specifically, increase in bursting in LHb^→DRN^ neurons is both sufficient and necessary for increasing anhedonia and anxiety‐like behaviors following long‐term exposure to AS. Additionally, while the increased bursting in all these LHb neuronal subpopulations contributes to an increase of despair‐like behaviors, the simultaneous inhibition of bursting in LHb^→DRN^, LHb^→VTA^, and LHb^→MnR^ neurons is required to reduce despair‐like behavior following exposure to AS. Finally, decreased bursting in distinct subpopulations of LHb neurons is essential for the therapeutic effects of BLT in mitigating distinct depressive‐like behaviors induced by long‐term exposure to AS.

## 2 Results

### 2.1 Long‐term Exposure to Aversive Stimuli Increases Burst Firing in LHb^→DRN^, LHb^→VTA^, and LHb^→MnR^ Neurons

It is well established that LHb neurons can directly innervate downstream aminergic reward centers, including the DRN, VTA, and MnR.^[^ ^3^, ^8^ ^]^ In line with these studies, we introduced an adenovirus encoding a green fluorescent protein (AAV2/9‐hSyn‐EGFP) into the LHb of C57BL/6 mice and observed dense innervation in the DRN, VTA, and MnR (Figure S1A, Supporting Information). To ascertain whether LHb^→DRN^, LHb^→VTA^, and LHb^→MnR^ neurons constitute largely separate circuits, we employed retrograde viral tracing, with each virus conjugated to a different fluorophore (either EGFP or mCherry). One conjugate was bilaterally injected into one target region, and the other fluorophore was bilaterally injected into another target region (Figure S1B,D,F, Supporting Information). Notably, there was minimal overlap among the three LHb populations (Figure S1C,E,G,Supporting Information). To further investigate whether distinct subpopulations of LHb neurons can project to more than one brain region simultaneously, we injected rAAV2/2‐Retro‐Cre into one target region and rAAV2/2‐Retro‐DIO‐Flp into another target region. Subsequently, we injected AAV2/9‐fDIO‐EYFP into the LHb (Figure S1H,J,L, Supporting Information). We found that LHb^→DRN^, LHb^→VTA^, and LHb^→MnR^ neurons rarely project to more than one brain region simultaneously (Figure S1H–M, Supporting Information). Together, our results suggest that the LHb^→DRN^, LHb^→VTA^, and LHb^→MnR^ projection pathways are anatomically distinct.

**Figure 1:**
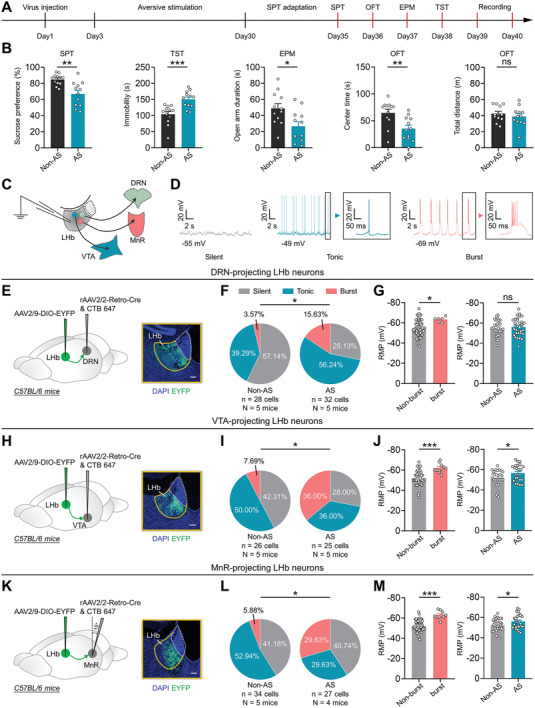
Long‐term exposure to aversive stimuli increases burst firing in LHb^→DRN^, LHb^→VTA^, and LHb^→MnR^ neurons. A) Schematic of the experimental design. B) Depressive‐like and anxiety‐like behaviors of mice in different experimental groups (Non‐AS: *n* = 12 mice; AS: N = 12 mice). Non‐AS, mice without exposure to AS; AS, mice that exposure to AS. Data presents Mean ± SEM; Two tail unpaired *t*‐test, ^*^, *p *< 0.05; ^**^, *p *< 0.01; ^***^, *p *< 0.001; ns, no significant difference. C) Scheme for whole cell patch‐clamp recording of LHb^→DRN^, LHb^→VTA^, and LHb^→MnR^ neurons. D) Representative traces showing spontaneous activity of silent, tonic firing, and burst firing LHb neurons. E–G) Scheme for specific infection of LHb^→DRN^ neurons with EYFP. Scale bar: 100 µm E); Pie charts indicate percentages of the three types of LHb^→DRN^ neurons in Non‐AS (*n* = 28 cells, N = 5 mice) and AS (*n* = 32 cells, N = 5 mice) groups. Data presents chi‐square test, *, *p *< 0.05 F); RMP of Non‐bursting LHb^→DRN^ neurons (*n* = 54 cells, N = 10 mice) and bursting LHb^→DRN^ neurons (*n* = 6 cells, N = 10 mice) (left) and RMP of the LHb^→DRN^ neurons in Non‐AS (*n* = 28 cells, N = 5 mice) and AS (*n* = 32 cells, N = 5 mice) groups (right), data presents Mean ± SEM; Two tail unpaired *t*‐test, ^*^, *p *< 0.05; ns, no significant difference G). All mice received DRN injection of rAAV2/2‐Retro‐Cre and CTB‐647, LHb injection of AAV2/9‐DIO‐EYFP. Non‐AS, mice without exposure to AS; AS, mice that exposure to AS. H‐J) Scheme for specific infection of LHb^→VTA^ neurons with EYFP. Scale bar: 100 µm (H); Pie charts indicate percentages of the three types of LHb^→VTA^ neurons in Non‐AS (*n* = 26 cells, N = 5 mice) and AS (*n* = 25 cells, N = 5 mice) groups. Data presents chi‐square test, ^*^, *p *< 0.05 (I); RMP of Non‐bursting LHb^→VTA^ neurons (*n* = 40 cells, N = 10 mice) and bursting LHb^→VTA^ neurons (*n* = 11 cells, N = 10 mice) (left), and RMP of the LHb^→VTA^ neurons in Non‐AS (*n* = 26 cells, N = 5 mice) and AS (*n* = 25 cells, N = 5 mice) groups (right), data presents Mean ± SEM; Two tail unpaired *t*‐test, ^*^, *p *< 0.05; ^***^, *p *< 0.001 J). All mice received VTA injection of rAAV2/2‐Retro‐Cre and CTB‐647, LHb injection of AAV2/9‐DIO‐EYFP. Non‐AS, mice without exposure to AS; AS, mice that exposure to AS. K–M) Scheme for specific infection of LHb^→MnR^ neurons with EYFP. Scale bar: 100 µm K); Pie charts indicate percentages of the three types of LHb^→MnR^ neurons in the Non‐AS (*n* = 34 cells, N = 5 mice), AS (*n* = 27 cells, N = 4 mice) groups. Data presents chi‐square test, ^*^, *p *< 0.05 L); RMP of the Non‐bursting LHb^→MnR^ neurons (*n* = 51 cells, N = 9 mice) and bursting LHb^→MnR^ neurons (*n* = 10 cells, N = 9 mice) (left), and RMP of LHb^→MnR^ neurons in Non‐AS (*n* = 34 cells, N = 5 mice) and AS (*n* = 27 cells, N = 4 mice) groups (right), data presents Mean ± SEM; Two tail unpaired *t*‐test, ^*^, *p *< 0.05; ^***^, *p *< 0.001 M). All mice received MnR injection of rAAV2/2‐Retro‐Cre and CTB‐647, LHb injection of AAV2/9‐DIO‐EYFP. Non‐AS, mice without exposure to AS; AS, mice that exposure to AS.

To investigate how the spike patterns of LHb^→DRN^, LHb^→VTA^, and LHb^→MnR^ neurons are altered during the pathological process of depression, we delivered the monosynaptic retrograde transport virus rAAV2/2‐Retro‐Cre into the DRN, VTA, or MnR, along with AAV2/9‐DIO‐EYFP into the LHb (**Figures** **1**E,H,K; S1N–P, Supporting Information). The mice were then subjected to long‐term exposure to AS (foot shock, 20 times/day; air puff, 20 times/day; fox urine, 30 min/day; physical restraint, 1 h/day, 28 days) (Figure 1A), a well‐established animal model of depression.^[^ ^9^ ^]^ We found that long‐term exposure to AS significantly increased depressive‐like behaviors tested in the sucrose preference test (SPT), tail suspension test (TST) and anxiety‐like behaviors tested in the elevated plus maze test (EPM) and open field test (OFT), without affecting the locomotor activities tested in the OFT (Figure 1B). Notably, patch‐clamp recordings of LHb^→DRN^, LHb^→VTA^, and LHb^→MnR^ neurons from these mice revealed a marked increase in burst firing and hyperpolarization of the resting membrane potential (RMP) of the bursting neurons (Figure 1C–M). Given that increased bursting in LHb neurons is associated with depressive‐like and anxiety‐like behaviors,^[^ ^6^ ^]^ these results suggest that the heightened bursting in LHb^→DRN^, LHb^→VTA^, and LHb^→MnR^ neurons might influence the depressive‐like and anxiety‐like behaviors induced by long‐term exposure to AS.

### 2.2 Burst Firing in LHb^→DRN^, LHb^→VTA^, and LHb^→MnR^ Neurons Mediate Distinct Depressive‐like Symptoms

**Figure 2:**
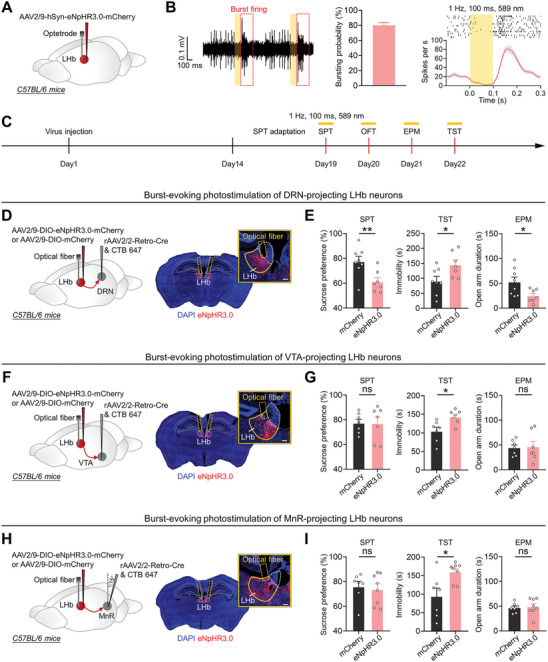
eNpHR3.0‐induced rebound bursting in LHb^→DRN^, LHb^→VTA^, and LHb^→MnR^ neurons mediates distinct depressive‐like symptoms. A) Scheme for infection of LHb neurons with eNpHR3.0 and the optetrode implantation in LHb. B) Representative traces showing rebound bursts elicited by pulsed yellow light (1 Hz, 100 ms, 589 nm) in the LHb in vivo from mice infected with eNpHR3.0 (right); Percentage of successfully induced bursts (*n* = 22 cells, N = 4 mice) (middle); Raster plots (top) and post‐stimulus time histogram (bottom) of an example LHb neuron responding to yellow light stimulation from in vivo optetrode recording (left). C) Schematic of the experimental design. D,E) Scheme for specific infection of LHb^→DRN^ neurons with eNpHR3.0 or mCherry. Scale bar: 100 µm D); Depressive‐like and anxiety‐like behaviors of mice in different experimental groups (mCherry: N = 8 mice; eNpHR3.0: N = 7 mice), data presents Mean ± SEM; Two tail unpaired *t*‐test, ^*^, *p *< 0.05; ^**^, *p *< 0.01. E); All mice received DRN injection of rAAV2/2‐Retro‐Cre and CTB‐647. mCherry, mice that received LHb injection of AAV2/9‐DIO‐mCherry; eNpHR3.0, mice that received LHb injection of AAV2/9‐DIO‐eNpHR3.0‐mCherry. F,G) Scheme for specific infection of LHb^→VTA^ neurons with eNpHR3.0 or mCherry. Scale bar: 100 µm F); Depressive‐like and anxiety‐like behaviors of mice in different experimental groups (mCherry: N = 7 mice; eNpHR3.0: N = 7 mice), data presents Mean ± SEM; Two tail unpaired *t*‐test, ^*^, *p *< 0.05; ns, no significant difference G). All mice received VTA injection of rAAV2/2‐Retro‐Cre and CTB‐647. mCherry, mice that received LHb injection of AAV2/9‐DIO‐mCherry; eNpHR3.0, mice that received LHb injection of AAV2/9‐DIO‐eNpHR3.0‐mCherry. H,I) Scheme for specific infection of LHb^→MnR^ neurons with eNpHR3.0 or mCherry. Scale bar: 100 µm H); Depressive‐like and anxiety‐like behaviors of mice in different experimental groups (mCherry: N = 7 mice; eNpHR3.0: N = 8 mice), data presents Mean ± SEM; Two tail unpaired *t*‐test, ^*^, *p *< 0.05; ns, no significant difference (I). All mice received MnR injection of rAAV2/2‐Retro‐Cre and CTB‐647. mCherry, mice that received LHb injection of AAV2/9‐DIO‐mCherry; eNpHR3.0, mice that received LHb injection of AAV2/9‐DIO‐eNpHR3.0‐mCherry.

To investigate the role of burst firing in LHb^→DRN^, LHb^→VTA^, and LHb^→MnR^ neurons in the development of depressive‐like symptoms, we employed an inhibitory opsin, eNpHR3.0 (1 Hz, 100 ms, 589 nm),^[^ ^6a^ ^]^ to induce rebound bursting in LHb^→DRN^, LHb^→VTA^, and LHb^→MnR^ neurons. This approach allowed us to examine the role of burst firing in the expression of depressive‐like symptoms.^[^ ^6a^ ^]^ Consistent with previous findings,^[^ ^6a^ ^]^ in vivo optetrode recordings demonstrated that the rebound burst protocol (1 Hz, 100 ms, 589 nm) consistently evoked burst firing in LHb neurons with a high success rate (**Figures** **2**A,B; S2A, Supporting Information). Notably, we found that for mice expressing eNpHR3.0 in LHb^→DRN^ neurons, 1 Hz yellow light photostimulation significantly reduced sucrose preference in the SPT, increased immobility in the TST and anxiety‐like behaviors in the EPM and OFT, without altering locomotion in the OFT (Figures 2C–E; S2B,C, Supporting information). In contrast, for mice expressing eNpHR3.0 in LHb^→VTA^ or LHb^→MnR^ neurons, 1 Hz yellow light photostimulation significantly increased immobility in the TST but had no significant effect on sucrose preference in the SPT, anxiety‐like behaviors in the EPM and OFT, or locomotion in the OFT (Figures 2C,F,G,H,I; S2D–G, Supporting Information). These results suggest that burst firing in LHb^→DRN^, LHb^→VTA^, and LHb^→MnR^ neurons may mediate distinct depressive‐like symptoms.

**Figure 3:**
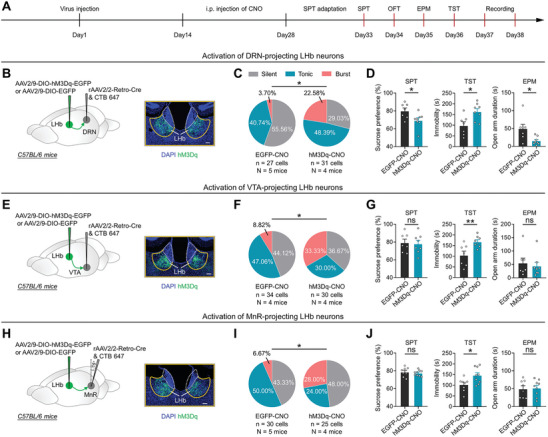
Long‐term activation induces burst firing in LHb^→DRN^, LHb^→VTA^, and LHb^→MnR^ neurons and mediates distinct depressive‐like symptoms. A) Schematic of the experimental design. B‐D) Scheme for specific infection of LHb^→DRN^ neurons with hM3Dq or EGFP. Scale bar: 100 µm (B); Pie charts indicate percentages of the three types of LHb^→DRN^ neurons in the EGFP‐CNO (*n* = 27 cells, N = 5 mice) and hM3Dq‐CNO (*n* = 31 cells, N = 4 mice) groups, data presents chi‐square test, ^*^, *p *< 0.05 C); Depressive‐like and anxiety‐like behaviors of mice in different experimental groups (EGFP‐CNO: N = 7 mice; hM3Dq‐CNO: N = 8 mice), data presents Mean ± SEM; Two tail unpaired *t*‐test, ^*^, *p *< 0.05. D). All mice received DRN injection of rAAV2/2‐Retro‐Cre and CTB‐647. EGFP‐CNO, mice that received LHb injection of AAV2/9‐DIO‐EGFP and i.p. injection of CNO (1 mg kg^−1^); hM3Dq‐CNO, mice that received LHb injection of AAV2/9‐DIO‐hM3Dq‐EGFP and i.p. injection of CNO (1 mg kg^−1^). E–G) Scheme for specific infection of LHb^→VTA^ neurons with hM3Dq or EGFP. Scale bar: 100 µm E); Pie charts indicate percentages of the three types of LHb^→VTA^ neurons in the EGFP‐CNO (*n* = 34 cells, N = 4 mice) and hM3Dq‐CNO (*n* = 30 cells, N = 4 mice) groups, data presents chi‐square test, ^*^, *p *< 0.05 F); Depressive‐like and anxiety‐like behaviors of mice in different experimental groups (EGFP‐CNO: N = 7 mice; hM3Dq‐CNO: N = 8 mice), data presents Mean ± SEM; Two tail unpaired *t*‐test (the SPT and the TST), Mann‐Whitney U test (the EPM), ^**^, *p *< 0.01; ns, no significant difference. G). All mice received VTA injection of rAAV2/2‐Retro‐Cre and CTB‐647. EGFP‐CNO, mice that received LHb injection of AAV2/9‐DIO‐EGFP and i.p. injection of CNO (1 mg kg^−1^); hM3Dq‐CNO, mice that received LHb injection of AAV2/9‐DIO‐hM3Dq‐EGFP and i.p. injection of CNO (1 mg kg^−1^). H–J) Scheme for specific infection of LHb^→MnR^ neurons with hM3Dq or EGFP. Scale bar: 100 µm H); Pie charts indicate percentages of the three types of LHb^→MnR^ neurons in the EGFP‐CNO (*n* = 30 cells, N = 5 mice) and hM3Dq‐CNO (*n* = 25 cells, N = 4 mice) groups, data presents chi‐square test, ^*^, *p *< 0.05 I); Depressive‐like and anxiety‐like behaviors of mice in different experimental groups (EGFP‐CNO: N = 8 mice; hM3Dq‐CNO: N = 9 mice), data presents Mean ± SEM; Two tail unpaired *t*‐test, ^*^, *p *< 0.05; ns, no significant difference J). All mice received MnR injection of rAAV2/2‐Retro‐Cre and CTB‐647. EGFP‐CNO, mice that received LHb injection of AAV2/9‐DIO‐EGFP and i.p. injection of CNO (1 mg kg^−1^); hM3Dq‐CNO, mice that received LHb injection of AAV2/9‐DIO‐hM3Dq‐EGFP and i.p. injection of CNO (1 mg kg^−1^).

Recent studies have suggested that burst firing in the LHb can be modulated by long‐term changes in LHb neuronal activity.^[^ ^3a^ ^]^ Therefore, we aimed to confirm the role of burst firing in LHb^→DRN^, LHb^→VTA^, and LHb^→MnR^ neurons during depressive‐like behaviors induced by long‐term activation of these specific LHb neurons. We delivered rAAV2/2‐Retro‐Cre into the DRN, VTA, or MnR, along with either the neuronal activator DREADD hM3Dq (AAV2/9‐DIO‐hM3Dq‐EGFP) or AAV2/9‐DIO‐EGFP as a control into the LHb (**Figures** **3**B,E,H; S3A,E,I, Supporting Information). LHb^→DRN^, LHb^→VTA^, and LHb^→MnR^ neurons were activated by daily intraperitoneal (i.p.) injections of Clozapine N‐oxide (CNO) (1 mg kg^−1^) for 14 days (Figures 3A; S3B,F,J, Supporting Information). We found that long‐term chemogenetic activation significantly increased bursting in LHb^→DRN^, LHb^→VTA^, and LHb^→MnR^ neurons, accompanied by hyperpolarization of the RMP in bursting neurons (Figures 3C,F,I; S3C,G,K, Supporting Information). Furthermore, long‐term activation of LHb^→DRN^ neurons significantly reduced sucrose preference in the SPT, increased immobility in the TST and anxiety‐like behaviors in the EPM and OFT, without altering locomotion in the OFT (Figures 3D; S3D, Supporting Information). In contrast, long‐term activation of both LHb^→VTA^ and LHb^→MnR^ neurons significantly increased immobility in the TST, but had no effect on sucrose preference in the SPT, anxiety‐like behaviors in the EPM and OFT, or locomotion in the OFT (Figures 3G,J; S3H,L, Supporting Information). These findings are consistent with the effects of light photostimulation (Figures 2E,G,I; S2C,E,G, Supporting Information), and suggest that increased bursting in LHb^→DRN^ neurons is associated with anhedonia, despair‐like and anxiety‐like behaviors in mice, while increased bursting in LHb^→VTA^ and LHb^→MnR^ neurons is associated with despair‐like behaviors in mice.

### 2.3 Long‐term Exposure to Aversive Stimuli Induces Depressive‐like Symptoms, Which Require Burst Firing in LHb^→DRN^, LHb^→VTA^, and LHb^→MnR^ Neurons

**Figure 4:**
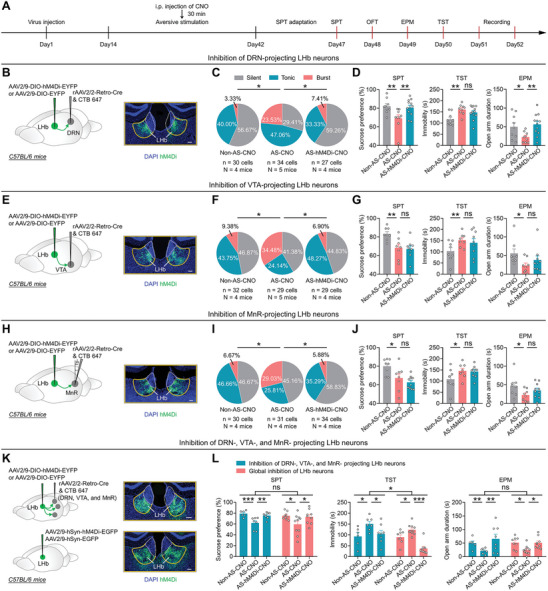
Long‐term exposure to aversive stimuli induces depressive‐like symptoms, which require burst firing in LHb^→DRN^, LHb^→VTA^, and LHb^→MnR^ neurons. A) Schematic of the experimental design. B–D) Scheme for specific infection of LHb^→DRN^ neurons with hM4Di or EYFP. Scale bar: 100 µm B); Pie charts indicate percentages of the three types of LHb^→DRN^ neurons in Non‐AS‐CNO (*n* = 30 cells, N = 4 mice), AS‐CNO (*n* = 34 cells, N = 5 mice) and AS‐hM4Di‐CNO (*n* = 27 cells, N = 4 mice) groups, data presents chi‐square test, ^*^, *p *< 0.05C); Depressive‐like and anxiety‐like behaviors of mice in different experimental groups (Non‐AS‐CNO: N = 9 mice; AS‐CNO: N = 9 mice; AS‐hM4Di‐CNO: N = 12 mice), data presents Mean ± SEM; One‐way ANOVA with post hoc LSD test, ^*^, *p *< 0.05; ^**^, *p *< 0.01; ns, no significant difference D). All mice received DRN injection of rAAV2/2‐Retro‐Cre and CTB‐647. Non‐AS‐CNO, mice that received LHb injection of AAV2/9‐DIO‐EYFP, i.p. injection of CNO (1 mg kg^−1^), and no exposure to AS; AS‐CNO, mice that received LHb injection of AAV2/9‐DIO‐EYFP, i.p. injection of CNO (1 mg kg^−1^), and exposure to AS; AS‐hM4Di‐CNO: mice that received LHb injection of AAV2/9‐DIO‐hM4Di‐EYFP, i.p. injection of CNO (1 mg kg^−1^), and exposure to AS. E–G) Scheme for specific infection of LHb^→VTA^ neurons with hM4Di or EYFP. Scale bar: 100 µm E); Pie charts indicate percentages of the three types of LHb^→VTA^ neurons in Non‐AS‐CNO (*n* = 32 cells, N = 4 mice), AS‐CNO (*n* = 29 cells, N = 5 mice), and AS‐hM4Di‐CNO (*n* = 29 cells, N = 4 mice) groups, data presents chi‐square test, ^*^, *p *< 0.05 F); Depressive‐like and anxiety‐like behaviors of mice in different experimental groups (Non‐AS‐CNO: N = 8 mice; AS‐CNO: N = 8 mice; AS‐hM4Di‐CNO: N = 9 mice), data presents Mean ± SEM; One‐way ANOVA with post hoc LSD test, ^*^, *p *< 0.05; ^**^, *p *< 0.01; ns, no significant difference G). All mice received VTA injection of rAAV2/2‐Retro‐Cre and CTB‐647. Non‐AS‐CNO, mice that received LHb injection of AAV2/9‐DIO‐EYFP, i.p. injection of CNO (1 mg kg^−1^), and no exposure to AS; AS‐CNO, mice that received LHb injection of AAV2/9‐DIO‐EYFP, i.p. injection of CNO (1 mg kg^−1^), and exposure to AS; AS‐hM4Di‐CNO, mice that received LHb injection of AAV2/9‐DIO‐hM4Di‐EYFP, i.p. injection of CNO (1 mg kg^−1^), and exposure to AS. H–J) Scheme for specific infection of LHb^→MnR^ neurons with hM4Di or EYFP. Scale bar: 100 µm H); Pie charts indicate percentages of the three types of LHb^→MnR^ neurons in Non‐AS‐CNO (*n* = 30 cells, N = 4 mice), AS‐CNO (*n* = 31 cells, N = 4 mice), and AS‐hM4Di‐CNO (*n* = 34 cells, N = 4 mice) groups, data presents chi‐square test, ^*^, *p *< 0.05 I); Depressive‐like and anxiety‐like behaviors of mice in different experimental groups (Non‐AS‐CNO: N = 8 mice; AS‐CNO: N = 8 mice; AS‐hM4Di‐CNO: N = 10 mice), data presents Mean ± SEM; One‐way ANOVA with post hoc LSD test, ^*^, *p *< 0.05; ns, no significant difference J). All mice received MnR injection of rAAV2/2‐Retro‐Cre and CTB‐647. Non‐AS‐CNO, mice that received LHb injection of AAV2/9‐DIO‐EYFP, i.p. injection of CNO (1 mg kg^−1^), and no exposure to AS; AS‐CNO, mice that received LHb injection of AAV2/9‐DIO‐EYFP, i.p. injection of CNO (1 mg kg^−1^), and exposure to AS; AS‐hM4Di‐CNO, mice that received LHb injection of AAV2/9‐DIO‐hM4Di‐EYFP, i.p. injection of CNO (1 mg kg^−1^), and exposure to AS. K,L) Scheme for simultaneous infection of LHb^→DRN^, LHb^→VTA^, and LHb^→MnR^ neurons with hM4Di or EYFP (top); Scheme for global infection of LHb neurons with hM4Di or EGFP. Scale bar: 100 µm (bottom) (K); Depressive‐like and anxiety‐like behaviors of mice in different experimental groups (blue label: Non‐AS‐CNO: N = 6 mice; AS‐CNO: N = 7 mice; AS‐hM4Di‐CNO: N = 8 mice) (red label: Non‐AS‐CNO: N = 8 mice; AS‐CNO: N = 10 mice; AS‐hM4Di‐CNO: N = 9 mice), data presents Mean ± SEM; One‐way ANOVA with post hoc LSD test and Two‐way ANOVA with Sidak's multiple‐comparisons test, ^*^, *p *< 0.05; ^**^, *p *<0.01; ^***^, *p *<0.001; ns, no significant difference L). For simultaneous inhibition of LHb^→DRN^, LHb^→VTA^, and LHb^→MnR^ neurons, all mice received DRN, VTA and MnR injection of rAAV2/2‐Retro‐Cre and CTB‐647, and LHb injection of AAV2/9‐DIO‐EYFP or AAV2/9‐DIO‐hM4Di‐EYFP. For global inhibition of LHb neurons, all mice received LHb injection of AAV2/9‐hSyn‐EGFP or AAV2/9‐hSyn‐hM4Di‐EGFP. Non‐AS‐CNO, mice with EYFP or EGFP expressed in LHb, received i.p. injection of CNO (1 mg kg^−1^), and no exposure to AS; AS‐CNO, mice with EYFP or EGFP expressed in LHb, received i.p. injection of CNO (1 mg kg^−1^), and exposure to AS; AS‐hM4Di‐CNO: mice with hM4Di expressed in LHb, received i.p. injection of CNO (1 mg kg^−1^), and exposure to AS.

To investigate the necessity of burst firing in LHb^→DRN^, LHb^→VTA^ or LHb^→MnR^ neurons for the depressive‐like symptoms induced by long‐term exposure to AS, we selectively inhibited these neurons during AS exposure. We injected rAAV2/2‐Retro‐Cre into the DRN, VTA, and MnR, respectively, and infected LHb neurons with either AAV2/9‐DIO‐hM4Di‐EYFP or AAV2/9‐DIO‐EYFP as a control (**Figures** **4**B,E,H; S4A,E,I, Supporting Information). Two weeks after virus injection, mice were exposed to long‐term AS, and the hM4Di‐expressing neurons were daily inhibited by i.p. injection of CNO (1 mg kg^−1^) (Figures 4A; S4B,F,J, Supporting Information). We found that chemogenetic inhibition successfully reduced bursting in LHb^→DRN^, LHb^→VTA^, and LHb^→MnR^ neurons in mice that were exposed to long‐term AS, and this change was accompanied by a reversal of the RMP hyperpolarization (Figures 4C,F,I; S4C,G,K, Supporting Information). Notably, inhibiting burst firing in LHb^→DRN^ neurons significantly ameliorated AS‐induced anhedonia in the SPT and anxiety‐like behaviors in the EPM and OFT but did not significantly affect despair‐like behaviors in the TST (Figures 4D; S4D, Supporting Information). In contrast, inhibiting burst firing in LHb^→VTA^ or LHb^→MnR^ neurons did not significantly affect AS‐induced anhedonia, despair‐like, and anxiety‐like behaviors (Figures 4G,J; S4H,L, Supporting Information).

Given that increased bursting in LHb^→DRN^, LHb^→VTA^, and LHb^→MnR^ neurons all have the potential to induce despair‐like behaviors (Figures 2E,G,I and 3D,G,J), and that chemogenetic inhibition of LHb neurons during AS exposure effectively reduced bursting induced by AS, we proceeded to examine whether simultaneous inhibition of LHb^→DRN^, LHb^→VTA^, and LHb^→MnR^ neurons could alleviate AS‐induced despair‐like behaviors. To test this possibility, we induced Cre recombinase expression in LHb^→DRN^, LHb^→VTA^, and LHb^→MnR^ neurons via concurrent injections of rAAV2/2‐Retro‐Cre into the DRN, VTA, and MnR (Figures 4K; S4M, Supporting Information). Subsequently, we infected LHb neurons with AAV2/9‐DIO‐hM4Di‐EYFP, while AAV2/9‐DIO‐EYFP was used as a control (Figure 4K). Our results revealed that simultaneous inhibition of LHb^→DRN^, LHb^→VTA^, and LHb^→MnR^ neurons significantly ameliorated AS‐induced anhedonia, despair‐like, and anxiety‐like behaviors (Figures 4L; S4Q, Supporting Information). Additionally, we found that globally inhibiting LHb neurons also significantly reduced AS‐induced anhedonia, despair‐like, and anxiety‐like behaviors (Figures 4K,L; S4N,Q, Supporting Information), as well as the AS‐induced increase in bursting and hyperpolarization of the RMP in LHb neurons (Figure S4O,P, Supporting Information). Interestingly, when comparing the effects of simultaneous inhibition of LHb^→DRN^, LHb^→VTA^, and LHb^→MnR^ neurons, global inhibition of LHb neurons resulted in a greater decrease in despair‐like behaviors in the TST (Figure 4L). This suggests that other downstream targets of the LHb may also be involved in the regulation of despair‐like behaviors. Taken together, these results suggest that AS‐induced anhedonia and anxiety‐like behaviors require burst firing in LHb^→DRN^ neurons, whereas AS‐induced despair‐like behaviors necessitate simultaneous burst firing in LHb^→DRN^, LHb^→VTA^, and LHb^→MnR^ neurons.

### 2.4 BLT Decreases Aversive Stimuli‐induced Burst Firing in LHb^→DRN^, LHb^→VTA^, and LHb^→MnR^ Neurons

**Figure 5:**
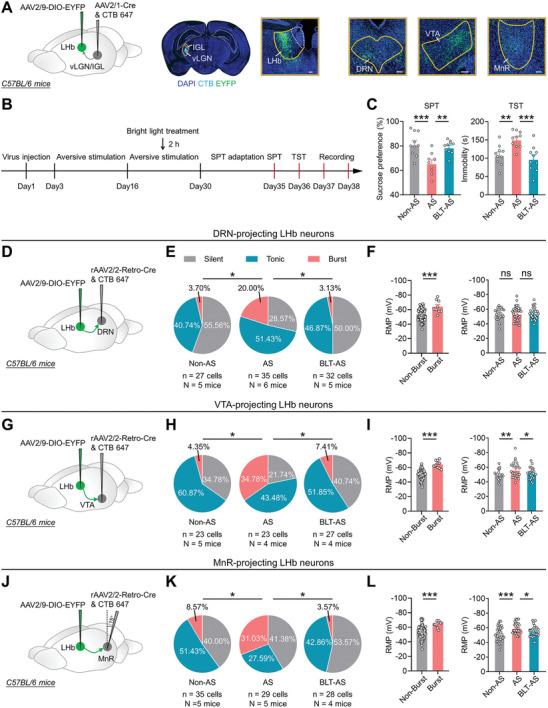
BLT decreases aversive stimuli‐induced burst firing in LHb^→DRN^, LHb^→VTA^, and LHb^→MnR^ neurons. A) Scheme for specific infection of LHb neurons receiving vLGN/IGL inputs with EYFP (right); Representative images of axons in the DRN, VTA and MnR from LHb postsynaptic neurons receiving vLGN/IGL inputs (left). Scale bar: 100 µm. B) Schematic of the experimental design. C) Depressive‐like behaviors of mice in different experimental groups (Non‐AS: N = 10 mice; AS: N = 10 mice; BLT‐AS: N = 10 mice), data presents Mean ± SEM; One‐way ANOVA with post hoc LSD test, ^**^, *p *< 0.01; ^***^, *p *< 0.001. Non‐AS, mice that without exposure to AS; AS, mice that exposure to AS; BLT‐AS, mice that exposure to AS and received BLT. D‐F) Scheme for specific infection of LHb^→DRN^ neurons with EYFP D); Pie charts indicate percentages of the three types of LHb^→DRN^ neurons in Non‐AS (*n* = 27 cells, N = 5 mice), AS (*n* = 35 cells, N = 6 mice) and BLT‐AS (*n* = 32 cells, N = 5 mice) groups, data presents chi‐square test, ^*^, *p *< 0.05 E); RMP of Non‐bursting LHb^→DRN^ neurons (*n* = 85 cells, N = 16 mice) and bursting LHb^→DRN^ neurons (*n* = 9 cells, N = 16 mice) (left), and RMP of the LHb^→DRN^ neurons in Non‐AS (*n* = 27 cells, N = 5 mice), AS (*n* = 35 cells, N = 6 mice) and BLT‐AS (*n* = 32 cells, N = 5 mice) groups (right), data presents Mean ± SEM; Two tail unpaired t‐test and One‐way ANOVA with post hoc LSD test, ^***^, *p *< 0.001; ns, no significant difference F). All mice received DRN injection of rAAV2/2‐Retro‐Cre and LHb injection of AAV2/9‐DIO‐EYFP. Non‐AS, mice without exposure to AS; AS, mice that exposure to AS; BLT‐AS, mice that exposure to AS and received BLT. G–I) Scheme for specific infection of LHb^→VTA^ neurons with EYFP G); Pie charts indicate percentages of the three types of LHb^→VTA^ neurons in Non‐AS (*n* = 23 cells, N = 5 mice), AS (*n* = 23 cells, N = 4 mice) and BLT‐AS (*n* = 27 cells, N = 4 mice) groups, data presents chi‐square test, ^*^, *p *< 0.05 (H); RMP of Non‐bursting LHb^→VTA^ neurons (*n* = 62 cells, N = 13 mice) and bursting LHb^→VTA^ neurons (*n* = 11 cells, N = 13 mice) (left), and RMP of the LHb^→VTA^ neurons in Non‐AS (*n* = 23 cells, N = 5 mice), AS (*n* = 23 cells, N = 4 mice) and BLT‐AS (*n* = 27 cells, N = 4 mice) groups (right), data presents Mean ± SEM; Two tail unpaired t‐test and One‐way ANOVA with post hoc LSD test, ^*^, *p *< 0.05; ^**^, *p *< 0.01; ^***^, *p *< 0.001 I). All mice received VTA injection of rAAV2/2‐Retro‐Cre and LHb injection of AAV2/9‐DIO‐EYFP. Non‐AS, mice without exposure to AS; AS, mice that exposure to AS; BLT‐AS, mice that exposure to AS and received BLT. J–L) Scheme for specific infection of LHb^→MnR^ neurons with EYFP (J); Pie charts indicate percentages of the three types of LHb^→MnR^ neurons in Non‐AS (*n* = 35 cells, N = 5 mice), AS (*n* = 29 cells, N = 5 mice) and BLT‐AS (*n* = 28 cells, N = 4 mice) groups, data presents chi‐square test, ^*^, *p *< 0.05 K); RMP of Non‐bursting LHb^→MnR^ neurons (*n* = 79 cells, N = 14 mice) and bursting LHb^→MnR^ neurons (*n* = 13 cells, N = 14 mice) (left), and RMP of the LHb^→MnR^ neurons in Non‐AS (*n* = 35 cells, N = 5 mice), AS (*n* = 29 cells, N = 5 mice) and BLT‐AS (*n* = 28 cells, N = 4 mice) groups (right), data presents Mean ± SEM; Two tail unpaired t‐test and One‐way ANOVA with post hoc LSD test, ^*^, *p *< 0.05; ^***^, *p *< 0.001 L). All mice received MnR injection of rAAV2/2‐Retro‐Cre and LHb injection of AAV2/9‐DIO‐EYFP. Non‐AS, mice without exposure to AS; AS, mice that exposure to AS; BLT‐AS, mice that exposure to AS and received BLT.

Considering our previous finding that BLT can reduce burst firing in the LHb and alleviate AS‐induced depressive‐like behaviors by activating LHb‐projecting GABAergic neurons in the vLGN/IGL,^[^ ^3a^ ^]^ we first investigated whether the DRN, VTA, and MnR were downstream targets of the LHb innervated by vLGN/IGL neurons. We injected the monosynaptic anterograde transport virus AAV2/1‐Cre into the vLGN/IGL and a Cre‐dependent virus encoding a yellow fluorescent protein (AAV2/9‐DIO‐EYFP) into the LHb of C57BL/6 mice (**Figure** **5**A). We found dense innervation in the DRN, VTA, and MnR (Figure 5A), suggesting that LHb neurons receiving direct vLGN/IGL inputs could further project to the DRN, VTA, and MnR. Subsequently, we assessed the impact of BLT on AS‐induced burst firing in LHb^→DRN^, LHb^→VTA^, and LHb^→MnR^ neurons. We injected rAAV2/2‐Retro‐Cre into the DRN, VTA, or MnR, respectively, and AAV2/9‐DIO‐EYFP into the LHb to label LHb^→DRN^, LHb^→VTA^, and LHb^→MnR^ neurons with EYFP (Figure 5D,G,J), and exposed the mice to long‐term AS. During the last 14 days of AS exposure, the mice were received 2 h of daily BLT (3000 lux) (Figure 5B). We found that BLT significantly reduced AS‐induced depressive‐like behaviors (Figure 5C), which is consistent with the findings of our previous study.^[^ ^3a^ ^]^ Furthermore, BLT reversed the AS‐induced increase in burst firing in LHb^→DRN^, LHb^→VTA^, and LHb^→MnR^ neurons (Figure 5E,H,K), along with the increased RMP (Figure 5F,I,L). These results collectively indicate that BLT effectively inhibits AS‐induced burst firing in LHb^→DRN^, LHb^→VTA^, and LHb^→MnR^ neurons.

### 2.5 The Antidepressant Effect of BLT Requires the Inhibition of Burst Firing in LHb^→DRN^, LHb^→VTA^, and LHb^→MnR^ Neurons

**Figure 6:**
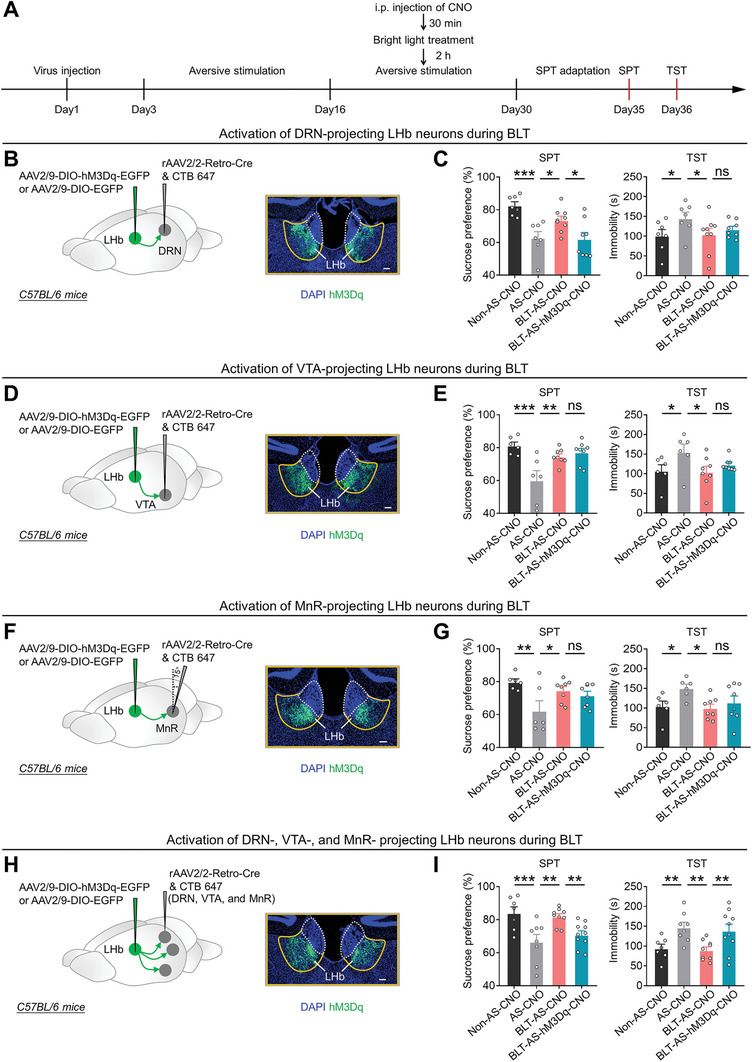
The antidepressant effect of BLT requires the inhibition of burst firing in LHb^→DRN^, LHb^→VTA^, and LHb^→MnR^ neurons. A) Schematic of the experimental design. B,C) Scheme for specific infection of LHb^→DRN^ neurons with hM3Dq or EGFP, Scale bar: 100 µm B); Depressive‐like behaviors of mice in different experimental groups (Non‐AS‐CNO: N = 7 mice; AS‐CNO: N = 7 mice; BLT‐AS‐CNO: N = 9 mice; BLT‐AS‐hM3Dq‐CNO: N = 8 mice), data presents Mean ± SEM; One‐way ANOVA with post hoc LSD test, ^*^, *p *< 0.05; ^***^, *p *< 0.001; ns, no significant difference C). All mice received DRN injection of rAAV2/2‐Retro‐Cre and CTB‐647. Non‐AS‐CNO, mice that received LHb injection of AAV2/9‐DIO‐EGFP, i.p. injection of CNO (1 mg kg^−1^), and no exposure to AS; AS‐CNO, mice that received LHb injection of AAV2/9‐DIO‐EGFP, i.p. injection of CNO (1 mg kg^−1^), and exposure to AS; BLT‐AS‐CNO, mice that received LHb injection of AAV2/9‐DIO‐EGFP, i.p. injection of CNO (1 mg kg^−1^), BLT, and exposure to AS; BLT‐AS‐hM3Dq‐CNO, mice that received LHb injection of AAV2/9‐DIO‐hM3Dq‐EGFP, i.p. injection of CNO (1 mg kg^−1^), BLT, and exposure to AS. D,E) Scheme for specific infection of LHb^→VTA^ neurons with hM3Dq or EGFP, Scale bar: 100 µm D); Depressive‐like behaviors of mice in different experimental groups (Non‐AS‐CNO: N = 6 mice; AS‐CNO: N = 6 mice; BLT‐AS‐CNO: N = 8 mice; BLT‐AS‐hM3Dq‐CNO: N = 8 mice), data presents Mean ± SEM; One‐way ANOVA with post hoc LSD test, ^*^, *p *< 0.05; ^**^, *p *< 0.01; ^***^, *p *< 0.001; ns, no significant difference E). All mice received VTA injection of rAAV2/2‐Retro‐Cre and CTB‐647. Non‐AS‐CNO, mice that received LHb injection of AAV2/9‐DIO‐EGFP, i.p. injection of CNO (1 mg kg^−1^), and no exposure to AS; AS‐CNO, mice that received LHb injection of AAV2/9‐DIO‐EGFP, i.p. injection of CNO (1 mg kg^−1^), and exposure to AS; BLT‐AS‐CNO, mice that received LHb injection of AAV2/9‐DIO‐EGFP, i.p. injection of CNO (1 mg kg^−1^), BLT, and exposure to AS; BLT‐AS‐hM3Dq‐CNO, mice that received LHb injection of AAV2/9‐DIO‐hM3Dq‐EGFP, i.p. injection of CNO (1 mg kg^−1^), BLT, and exposure to AS. F,G) Scheme for specific infection of LHb^→MnR^ neurons with hM3Dq or EGFP, Scale bar: 100 µm F); Depressive‐like behaviors of mice in different experimental groups (Non‐AS‐CNO: N = 6 mice; AS‐CNO: N = 6 mice; BLT‐AS‐CNO: N = 8 mice; BLT‐AS‐hM3Dq‐CNO: N = 8 mice), data presents Mean ± SEM; One‐way ANOVA with post hoc LSD test, ^*^, *p *< 0.05; ^**^, *p *< 0.01; ns, no significant difference G). All mice received MnR injection of rAAV2/2‐Retro‐Cre and CTB‐647. Non‐AS‐CNO, mice that received LHb injection of AAV2/9‐DIO‐EGFP, i.p. injection of CNO (1 mg kg^−1^), and no exposure to AS; AS‐CNO, mice that received LHb injection of AAV2/9‐DIO‐EGFP, i.p. injection of CNO (1 mg kg^−1^), and exposure to AS; BLT‐AS‐CNO, mice that received LHb injection of AAV2/9‐DIO‐EGFP, i.p. injection of CNO (1 mg kg^−1^), BLT, and exposure to AS; BLT‐AS‐hM3Dq‐CNO, mice that received LHb injection of AAV2/9‐DIO‐hM3Dq‐EGFP, i.p. injection of CNO (1 mg kg^−1^), BLT, and exposure to AS. H‐I) Scheme for simultaneous infection of LHb^→DRN^, LHb^→VTA^, and LHb^→MnR^ neurons with hM3Dq or EGFP. Scale bar: 100 µm (H); Depressive‐like behaviors of mice in different experimental groups (Non‐AS‐CNO: N = 7 mice; AS‐CNO: N = 8 mice; BLT‐AS‐CNO: N = 9 mice; BLT‐AS‐hM3Dq‐CNO: N = 10 mice), data presents Mean ± SEM; One‐way ANOVA with post hoc LSD test, ^**^, *p *< 0.01; ^***^, *p *< 0.001 I). All mice received DRN, VTA, and MnR injection of rAAV2/2‐Retro‐Cre and CTB‐647. Non‐AS‐CNO, mice that received LHb injection of AAV2/9‐DIO‐EGFP, i.p. injection of CNO (1 mg kg^−1^), and no exposure to AS; AS‐CNO, mice that received LHb injection of AAV2/9‐DIO‐EGFP, i.p. injection of CNO (1 mg kg^−1^), and exposure to AS; BLT‐AS‐CNO, mice that received LHb injection of AAV2/9‐DIO‐EGFP, i.p. injection of CNO (1 mg kg^−1^), BLT, and exposure to AS; BLT‐AS‐hM3Dq‐CNO, mice that received LHb injection of AAV2/9‐DIO‐hM3Dq‐EGFP, i.p. injection of CNO (1 mg kg^−1^), BLT, and exposure to AS.

To further investigate the role of burst firing in LHb^→DRN^, LHb^→VTA^, and LHb^→MnR^ neurons in the antidepressant effect of BLT, we injected rAAV2/2‐Retro‐Cre into bilateral DRN, VTA or MnR regions, respectively, and injected AAV2/9‐DIO‐hM3Dq‐EGFP or AAV2/9‐DIO‐EGFP into bilateral LHb (**Figures** **6**B,D,F; S5A–C, Supporting Information). LHb^→DRN^, LHb^→VTA^, and LHb^→MnR^ neurons were chemogenetically activated by daily i.p. injection of CNO (1 mg kg^−1^) during BLT (3000 lux, 2 h/day) (Figure 6A). We found that activation of LHb^→DRN^ neurons negated the anti‐anhedonic effect of BLT in mice that received long‐term AS exposure (Figure 6C). However, activation of LHb^→DRN^, LHb^→VTA^, and LHb^→MnR^ neurons individually did not negate the anti‐despair effect of BLT (Figure 6C,E,G). To further determine whether the simultaneous decrease in burst firing in these LHb neurons is required for the anti‐despair effect of BLT, we expressed Cre recombinase in LHb neurons via simultaneous injection of rAAV2/2‐Retro‐Cre into DRN, VTA, and MnR and infected LHb neurons with AAV2/9‐DIO‐hM3Dq‐EGFP or AAV2/9‐DIO‐EGFP as a control (Figures 6H; S5D, Supporting Information). We found that simultaneous activation of LHb^→DRN^, LHb^→VTA^, and LHb^→MnR^ neurons was sufficient to nullify both the anti‐anhedonic and the anti‐despair effects of BLT (Figure 6I). Taken together, these findings suggest that the inhibition of burst firing in LHb^→DRN^ neurons is necessary for the anti‐anhedonic effect of BLT, while the simultaneous inhibition of burst firing in LHb^→DRN^, LHb^→VTA^, and LHb^→MnR^ neurons is necessary for the anti‐despair effect of BLT.

## 3 Discussion

In this study, we demonstrated that long‐term exposure to AS increased burst firing in LHb^→DRN^, LHb^→VTA^, and LHb^→MnR^ neurons. Furthermore, through optogenetic and chemogenetic manipulations, we found that burst firing in LHb^→DRN^, LHb^→VTA^, and LHb^→MnR^ neurons mediated distinct depressive‐like symptoms, while decreased bursting in these LHb neuronal subpopulations mediated the distinct antidepressant effects of BLT. Our results underscore the significance of burst firing in output‐defined, parallel LHb circuits in the development of AS‐induced depressive‐like symptoms and the therapeutic potential of BLT in this context.

LHb neurons have direct connections with aminergic reward centers, which play crucial roles in mood‐related disorders.^[^ ^3^, ^8^, ^10^ ^]^ Consistent with these findings, we observed robust projections from LHb neurons to the VTA, DRN, and MnR. Furthermore, retrograde viral tracing revealed minimal overlap and rare projections to more than one brain region simultaneously among LHb^→DRN^, LHb^→VTA^, and LHb^→MnR^ neurons, suggesting that these subpopulations form largely separate circuits.^[^ ^11^ ^]^ Given that recent studies by using high‐throughput single‐cell transcriptional profiling have identified transcriptionally distinct neuronal cell types within the LHb that had distinct downstream targets,^[^ ^12^ ^]^ suggesting that LHb^→DRN^, LHb^→VTA^ and LHb^→MnR^ neurons may exhibit heterogeneity at the RNA level. Future studies will be necessary to establish the nature of this heterogeneity and to understand how it contributes to the diverse functions of LHb neurons in the regulation of mood‐related behaviors.

The LHb hosts primarily glutamatergic neurons, but it inhibits the downstream aminergic reward centers including the VTA, DRN, and MnR.^[^ ^3^, ^13^ ^]^ Although it is well‐established that long‐term AS exposure can increase burst firing in LHb neurons, the changes in the spike patterns of LHb neurons projecting to the VTA, DRN, and MnR during depression remain unclear. Our study revealed that prolonged AS exposure significantly increased bursting in LHb^→DRN^, LHb^→VTA^, and LHb^→MnR^ neurons. Given that burst firing can enhance synaptic transmission and facilitate synaptic plasticity,^[^ ^14^ ^]^ and that LHb neurons can directly project to the GABAergic interneurons within the VTA, DRN, and MnR,^[^ ^12^, ^15^ ^]^ it is plausible that enhanced bursting in LHb^→DRN^, LHb^→VTA^, and LHb^→MnR^ neurons may provide stronger outputs to inhibitory interneurons within the VTA, DRN, and MnR, thereby suppressing the activity of dopaminergic, serotonergic, or glutamatergic neuron activity in these brain regions. This hypothesis warrants further investigation in future studies.

Through the utilization of optogenetic and chemogenetic manipulations to induce burst firing, our data indicate that burst firing in distinct projecting LHb neurons mediates different depressive‐like symptoms. Notably, we are the first to observe that increased bursting in LHb^→DRN^ neurons mediates AS‐induced anhedonia and anxiety‐like behaviors, aligning with previous research indicating the involvement of LHb^→DRN^ neurons in reward‐seeking and the role of the DRN in anxiety‐like behaviors.^[^ ^16^ ^]^ Furthermore, while increased bursting in LHb neurons projecting to the VTA, DRN or MnR promote despair‐like behaviors, our findings demonstrate that simultaneous inhibition, rather than staggered inhibition, of burst firing in LHb^→DRN^, LHb^→VTA^, and LHb^→MnR^ neurons significantly ameliorates AS‐induced despair‐like behaviors. This finding is consistent with previous findings on the roles of LHb^→DRN^, LHb^→VTA^, and LHb^→MnR^ neurons in promoting despair‐like behaviors,^[^ ^16^, ^17^ ^]^ and our results suggest that AS‐induced despair‐like behaviors require simultaneous inhibition of burst firing in LHb^→DRN^, LHb^→VTA^, and LHb^→MnR^ neurons. Additionally, we found that global inhibition of burst firing in LHb neurons was associated with a greater decrease in immobility than the inhibition of only specific LHb neurons. Since LHb neurons also project to the rostromedial tegmental nucleus (RMTg), and inhibiting LHb^→RMTg^ neurons can decrease immobility in the FST,^[^ ^18^ ^]^ suggesting that other downstream targets of the LHb may contribute to despair‐like behaviors, such as the RMTg.

In our previous study, we found that BLT reduced bursting in LHb neurons and reversed AS‐induced depressive‐like behaviors via the vLGN/IGL‐LHb pathway.^[^ ^3a^ ^]^ However, how the spike patterns of downstream projecting LHb neurons are affected by BLT and how these neurons mediate the antidepressant effects of BLT remain unclear. Consistent with our previous studies, our virus‐tracing data indicate that LHb postsynaptic neurons receive input from the vLGN/IGL and project to the VTA, DRN, and MnR. Furthermore, we demonstrated that BLT is sufficient to reverse the AS‐induced increase in burst firing in LHb^→DRN^, LHb^→VTA^, and LHb^→MnR^ neurons, possibly mediated by BLT's enhancement of GABAergic input to the LHb. Indeed, previous findings suggest that burst firing in the LHb can be modulated by network synaptic inputs.^[^ ^6a^ ^]^ Moreover, our data revealed that the inhibition of burst firing in LHb^→DRN^ neurons is required for the anti‐anhedonic effect of BLT, while the simultaneous inhibition of burst firing in LHb^→DRN^, LHb^→VTA^, and LHb^→MnR^ neurons is necessary for the anti‐despair effect of BLT. These findings highlight that the anti‐anhedonic and anti‐despair effects of BLT are achieved by reducing burst firing in distinct LHb neurons.

Our study revealed that the inhibition of burst firing in LHb^→DRN^ neurons reduced AS‐induced anxiety‐like behaviors. It is noteworthy that BLT tends to have anxiogenic effects in our previous study.^[^ ^3a^ ^]^ Consequently, it is reasonable to infer that the anxiogenic effects of BLT are mediated by brain regions rather than the LHb. Supporting this hypothesis, our study and other works show that BLT can increase amygdala activation.^[^ ^3^, ^19^ ^]^ Therefore, we did not assess the effect of BLT on anxiety‐like behaviors in this study.

In summary, our study offers compelling evidence that burst firing in output‐defined, parallel LHb circuits underlies the pathology of AS‐induced depressive‐like symptoms and the antidepressant effect of BLT. These findings improve our understanding of the mechanisms of both depression and the antidepressant effects of BLT.

## 4 Experimental Section

### Animals

Male C57BL/6 mice of adult age (6–8 weeks) were utilized for all experiments. The mice were kept on a 12 h: 12 h light‐dark cycle (lights on at 7 AM) with ad libitum access to food and water. The mice were assigned to the experimental and control groups randomly. During behavioral trials, the experimenters were blinded to the experimental group, and the order of testing was randomized. Mouse care and experiments were performed according to the guidelines for the Care and Use of Laboratory Animals of the National Institutes of Health. All experiments were approved by the Jinan University Institutional Animal Care and Use Committee (20210220‐12).

### Surgery and Intracranial Injection

Mice were anesthetized with Avertin (Avertin, 13 µL g^−1^, i.p.) and put in a stereotaxic device (RWD, Shenzhen, China) for surgery. To avoid corneal dryness, erythromycin eye ointment was used, and a heating pad was utilized to maintain body temperature at 37 °C. A small craniotomy hole was created using a dental drill (OmniDrill35, WPI, Sarasota, FL), and injections were administered using a micropipette connected to a Nanoliter Injector (NANOLITER 2010, WPI, Sarasota, FL) and its controller (Micro4, WPI, Sarasota, FL) at an injection rate of 0.1 µL min^−1^ to avoid potential damage to local brain tissue.

To infect the LHb neurons with EGFP or hM4Di‐EGFP or eNpHR3.0‐mCherry, AAV2/9‐hSyn‐ EGFP (virus titer: 3.5 × 10^12^ GC mL^−1^, 0.1 µL/injection; Taitool BioScience Co. Ltd., Shanghai, China) or AAV2/9‐hSyn‐hM4Di‐EGFP (virus titer: 3.5 × 10^12^ GC mL^−1^, 0.1 µL/injection; Taitool BioScience Co. Ltd., Shanghai, China) or AAV2/9‐hSyn‐eNpHR3.0‐mCherry (virus titer: 3.5 × 10^12^ GC/mL, 0.1 µL/injection; Taitool BioScience Co. Ltd., Shanghai, China) was injected into the LHb (*AP*: −1.6 mm; *ML*: ±0.3 mm; *DV*: −3.05 mm).

To label LHb^→DRN^, LHb^→VTA^, or LHb^→MnR^ neurons with EGFP or mCherry, rAAV2/2‐Retro‐mCherry (virus titer: 3 × 10^12^ GC mL^−1^, 0.15 µL/injection; Taitool BioScience Co. Ltd., Shanghai, China) or rAAV2/2‐Retro‐EGFP (virus titer: 3 × 10^12^ GC mL^−1^, 0.15 µL/injection; Taitool BioScience Co. Ltd., Shanghai, China) were injected into the DRN (*AP*: −4.50 mm; *ML*: 0 mm; *DV*: −2.5 mm), VTA (*AP*: −2.60 mm; *ML*: ±0.25 mm; *DV*: −4.5 mm), or MnR (*AP*: −5.35 mm; *ML*: 0 mm; *DV*: −4.50 mm, with a 15° angle).

To specifically infect LHb neurons with fDIO‐EYFP, AAV2/9‐fDIO‐EYFP (virus titer: 3.5 × 10^12^ GC mL^−1^, 0.1 µL/injection; Taitool BioScience Co. Ltd., Shanghai, China) was injected into LHb, rAAV2/2‐Retro‐Cre (virus titer: 3 × 10^12^ GC mL^−1^, 0.1 µL/injection; Taitool BioScience Co. Ltd., Shanghai, China) and rAAV2/2‐Retro‐DIO‐Flp (virus titer: 3 × 10^12^ GC mL^−1^, 0.1 µL/injection; BrainVTA Co. Ltd., Wuhan, China) were injected into DRN and MnR; MnR and VTA; VTA and DRN, respectively.

To specifically infect the postsynaptic LHb neurons from vLGN/IGL with EYFP, AAV2/1‐Cre (virus titer: 1.5 × 10^12^ GC mL^−1^; 0.2 µL/injection; Taitool BioScience Co. Ltd., Shanghai, China) and Alexa Fluor conjugated cholera toxin subunit B (CTB‐647, 0.02 µL/injection, Invitrogen Inc., Grand Island, NY) were injected into the vLGN/IGL (*AP*: −2.2 mm; *M*L: ± 2.5 mm; *DV*: −3.2 mm), and AAV2/9‐DIO‐EYFP (virus titer: 3.5 × 10^12^ GC mL^−1^, 0.1 µL/injection; Taitool BioScience Co. Ltd., Shanghai, China) was injected into the LHb.

To specifically infect LHb^→DRN^, LHb^→VTA^, or LHb^→MnR^ neurons with EGFP or hM3Dq‐EGFP or EYFP or hM4Di‐EYFP or mCherry or eNpHR3.0‐mCherry, rAAV2/2‐Retro‐Cre (virus titer: 3 × 10^12^ GC mL^−1^, 0.15 µL/injection; Taitool BioScience Co. Ltd., Shanghai, China) and Alexa Fluor conjugated cholera toxin subunit B (CTB‐647, 0.02 µL/injection, Invitrogen Inc., Grand Island, NY) were injected into the DRN, VTA, or MnR of C57BL/6 mice, and AAV2/9‐DIO‐EGFP (virus titer: 3.5 × 10^12^ GC mL^−1^, 0.1 µL/injection; Taitool BioScience Co. Ltd., Shanghai, China) or AAV2/9‐DIO‐EYFP or AAV2/9‐DIO‐hM3Dq‐EGFP (virus titer: 3.5 × 10^12^ GC mL^−1^, 0.1 µL/injection; Taitool BioScience Co. Ltd., Shanghai, China) or AAV2/9‐DIO‐hM4Di‐EYFP (virus titer: 3.5 × 10^12^ GC mL^−1^, 0.1 µL/injection; BrainCase Co. Ltd., Wuhan, China) or AAV2/9‐DIO‐mCherry (virus titer: 3.5 × 10^12^ GC mL^−1^, 0.1 µL/injection; Taitool BioScience Co. Ltd., Shanghai, China) or AAV2/9‐DIO‐eNpHR3.0‐mCherry (virus titer: 3.5 × 10^12^ GC mL^−1^, 0.1 µL/injection; Taitool BioScience Co. Ltd., Shanghai, China) was injected into the LHb.

### Injection Site Verification

Deeply anesthetized mice were perfused transcardially with 0.9% saline and then 4% paraformaldehyde in 0.1 m phosphate‐buffered saline (PBS). The brain was extracted and postfixed overnight at 4 °C with 4% PFA before being transferred to 30% sucrose and cryosectioned (CM1900, Leica Microsystems, Bannockburn, IL). To confirm the injection locations, a series of 40 µm coronal sections were collected. With a fluorescence microscope (Zeiss, Axioimager Z2 microscope), coronal brain slices were analyzed to determine the injection sites of viruses expressing fluorescent proteins (e.g., AAV2/9‐DIO‐hM3Dq‐EYFP and AAV2/9‐hSyn‐hM4Di‐EGFP). To visualize the injection sites of viruses that did not encode fluorescent protein (such as rAAV2/2‐Retro‐Cre and AAV2/1‐Cre), Alexa Fluor 647‐conjugated cholera toxin subunit B (CTB‐647) (0.02 µL /injection) was co‐injected into the targeted regions along with the viruses. Only mice with confirmed injection sites were used for study.

### Physiological Recordings from Brain Slices

For preparation of brain slices, mice were deeply anesthetized with isoflurane, and coronal sections containing LHb (250 µm thick) were cut using a vibratome (VT1200S; Leica Microsystems) in ice‐cold oxygenated modified artificial cerebrospinal fluid (ACSF) containing (in mM): 220 sucrose, 2.5 KCl, 1.3 CaCl_2_, 2.5 MgSO_4_, 1 NaH_2_PO_4_, 26 NaHCO_3_, and 10 glucose. Coronal LHb slices were then transferred to a storage chamber containing normal ACSF, which was composed of (in mM) 120 NaCl, 2.5 KCl, 1.2 NaH_2_PO_4_, 2.0 CaCl_2_, 2.0 MgSO_4_, 26 NaHCO_3_, and 10 glucose, for a 30 min recovery period at 34 °C and maintained at room temperature (25±1 °C) for at least 1 h prior to recording. All the solutions were saturated with 95% O_2_/5% CO_2_ (vol/vol).

Electrodes were filled with K^+^‐based solution containing (in mM) (130 K‐gluconate, 20 KCl, 10 HEPES buffer, 4 Mg‐ATP, 0.3 Na‐GTP, 10 disodium phosphocreatine and 0.2 EGTA, pH 7.2 with KOH, 280–320 mOsm). To determine the firing pattern and the RMP of LHb neurons, the spontaneous firing was recorded under current‐clamp at resting conditions (I = 0 pA) for 2 min. LHb neurons were classified as silent, tonic, or burst based on their firing patterns. Silent cells exhibited no spike activity during recording, tonic cells spontaneously generated tonic trains of action potentials at frequencies ranging from 0.1 and 10 Hz, occasionally up to 10–20 Hz, and burst‐firing cells produced clusters of spikes with a progressively declining intra‐burst firing frequency. The RMP was determined during the silent period of the three types of spontaneous activity. All recording was performed with a Multiclamp 700B amplifier (Molecular Devices). Traces were 2 kHz low‐pass filtered and digitized at 10 kHz. Pipette resistance varied between 4 and 6 MΩ. Basic electrophysiological properties were recorded when steady whole‐cell recordings could be obtained with an access resistance < 25 MΩ. Clampfit 10.0 software was used to conduct offline data analysis (Molecular Devices).

### Immunohistochemistry

All animals were anesthetized (avertin, 13 µL g^−1^, i.p.) and perfused intracardially with 0.9% saline followed by 4% PFA in PBS. Brains were carefully removed. All sections were rinsed in 0.1 m PBS and cover‐slipped in anti‐fading aqueous mounting medium with DAPI (EMS, Hatfield, PA).

### Image Analysis

Using a Zeiss 700 confocal microscope with a 5x or 20x objective or a 40x oil immersion objective, section samples were photographed. In order to recreate the 3D structure of injected or virus‐labeled cells, optical sections were acquired at intervals of 0.2 µm. Using ImageJ and Photoshop CS5 (Adobe Corp., San Jose, California, USA), each stack of optical sections was assembled and projected to a 0°X–Y plane and a 90°Y–Z plane to construct a 3D reconstruction of the cell. Contrast and brightness were adjusted, and the red‐green images were altered to magenta‐green.

### Behavioral Paradigms

All behavioral tests were conducted during the light phase (7 AM–7 PM). Investigators were blinded to the experimental groups during the scoring.

### Behavioral Paradigms—Foot Shock Exposure

Mice were put inside an acrylic box (25 cm × 25 cm × 40 cm) equipped with a metal grid floor. Foot shocks (20 times/day, 1 mA, 500 ms) were administered randomly with inter‐trial intervals of 15, 20 or 30 s to establish a depressive‐like and anxiety‐like mouse model. Mice in the control group were kept in the same acrylic box but were not subjected to the stimulation of foot shocks.

### Behavioral Paradigms–Air Puff Exposure

Mice were housed in their home cages, and air puffs (20 times/day) were randomly administered to the mice's faces with inter‐trial intervals of 10–15 s to establish a depressive‐like and anxiety‐like mouse model.

### Behavioral Paradigms–Fox Urine Exposure

Mice were housed in a transparent plastic container (25 cm × 10 cm × 10 cm, with holes) containing 4 cotton balls soaked with red fox urine‐for 30 min/day to establish a depressive‐like and anxiety‐like mouse model. Instead of fox urine, 2 mL of water for the control group was added.

### Behavioral Paradigms–Physical Restraint

Mice were restrained for 1 h/day in a plastic restrainer to establish a depressive‐like and anxiety‐like mouse model. The mice were returned to their own cages after restraint treatment.

### Behavioral Paradigms–Bright Light Treatment (BLT)

The mice in both the control and BLT groups were housed in their home cages, which were positioned on various shelves of a custom‐designed light cabinet. Mice were housed at room temperature with ad libitum access to food and water. Cool LED lights (UV‐free) with adjustable brightness were placed at the top of each shelf of the cabinet so that the brightness on each shelf could be manually controlled (the light intensity was determined by averaging the values from the top and the four sides of the cage). The animals in the control group were housed with a 12 h:12 h light/dark cycle with lights on from 7 AM – 7 PM (≈200 lux white ambient illumination). The animals in the BLT group were likewise housed under a 12 h:12 h light/dark cycle with lights on from 7–7 PM (≈200 lux white ambient illumination) except for during BLT (3 000 lux white ambient illumination between 8 and 10 AM). After housing in the light cabinet, all animals were subjected to behavioral tests as detailed below.

### Behavioral Paradigms–Sucrose Preference Test (SPT)

Mice were tested for preference for a 2% sucrose solution (Sucrose, Sigma–Aldrich) relative to plain water. Each mouse was housed singly during the 5‐days test period. In the first two days, mice were given two bottles of water. The next two days, water bottles were replaced into two bottles of 2% sucrose solution. After 4 days' adaptation, mice were water‐deprived for 24 h. Sucrose preference test was performed at dim light environment for 2 h, and mice were given one bottle of 2% sucrose solution and one bottle of plain water. The positions of the two bottles were switched after 1 h. The amounts of sucrose and water consumed were recorded. During experiments involving optogenetic manipulation, mice were subjected to a 30 min test. Light stimulation (589 nm yellow light laser, 1 Hz, 100 ms pulse) was delivered throughout the entire test, and the amounts of sucrose and water consumed were recorded. Food was available ad libitum prior to the SPT. The preference for the sucrose solution was determined as the percentage of sucrose solution ingested relative to the total intake.

### Behavioral Paradigms–Open Field Test (OFT)

The OFT was conducted to quantify anxiety‐related behavior as well as motor activity. The mice were placed in a plastic box (50 cm length × 50 cm width × 40 cm height) in a room with dim light and permitted to explore the arena for 15 min. With an infrared camera positioned above the cage, animal movement was captured. During experiments involving optogenetic manipulation, light stimulation (using a 589 nm yellow light laser, 1 Hz, 100 ms pulse) was delivered in the last 10 min of the test. Locomotion and time spent in the center during the last 10 min were measured using Ethovision XT software. After each test, the box was cleaned with a paper towel soaked in 50% ethanol and completely dried.

### Behavioral Paradigms–Elevated Plus Maze (EPM) Test

Anxiety‐like behavior is measured using the EPM test. The apparatus for the EPM consisted of two parts: two opposing open arms (44 cm length × 12 cm width) and two closed arms (44 cm length × 12 cm width), which were connected by a central zone (12 cm length × 12 cm width). The entire device was 50 cm above the ground. Animal was placed in the center zone facing an open arm and was allowed to freely explore the arena during a 6 min test session. The time spent in the open arm during last 4 min was measured using Ethovision XT software. During the experiment with optogenetic manipulation, light stimulation (589 nm yellow light laser, 1 Hz, 100 ms pulse) was delivered in the last 4 min of the test. The time spent in the open arm during this period was measured. After each test, the box was carefully cleaned using a paper towel dipped in 50% ethanol.

### Behavioral Paradigms–Tail Suspension Test (TST)

TST was used to assess the level of despair‐like behavior. In this procedure, the tails of mice were suffixed to a horizontal bar using adhesive tape for a duration of 6 min, and the immobility time was documented in the last 4 min of the test. During experiments involving optogenetic manipulation, light stimulation (589 nm yellow light laser, 1 Hz, 100 ms pulse) was delivered in the last 4 min of the test.

### Chemogenetic Manipulation

Each day at 9:00 AM, the designer drug CNO (1 mg kg^−1^, i.p.; C0832, Sigma–Aldrich, St. Louis, MO) was given to activate the hM3Dq‐expressed LHb neurons or inhibit the hM4Di‐expressed LHb neurons (Figure 3A). CNO was given 30 min before being exposed to AS (Figure 4A) or BLT (7:30 AM, Figure 6A).

### Optogenetic Manipulation

For optic fibre implantation, a 200 µm fiber‐optic cannula (Plastics one) was positioned 0.5 mm above the bilateral LHb and secured onto the skull using dental cement. During the behavioral tests, mice expressing eNpHR3.0 or mCherry received optogenetic manipulation (589 nm yellow light laser, 1 Hz, 100 ms pulse). The light intensity was calibrated to be 10 mW, ≈3.5 mm away from the tip of the optic patch cable with a ceramic sleeve.

### In Vivo Optetrode Recordings

To verify the effects of optogenetic manipulation on burst‐evoking, mice with eNpHR3.0 expression and a custom‐made screw‐driven optetrode implanted into the LHb were accepted optetrode recording. The optetrode consisted of an optical fiber and 16 single recording electrodes. During optical manipulation, the optetrode was connected to a 589 nm laser (Inper Technology Co. Ltd., Hangzhou, China) and it was set lower gradually for each unit recording. 100 ms light pulses at 1 Hz were delivered to evoke rebound bursting, and the light intensity (10 mW) was the same as behavioral tests. Data were acquired on NeuroStudio (Shanghai Kuo Yun Instruments Co. Ltd., Shanghai, China) and the recording signal was sampled at 30 kHz and band‐pass filtered at 300–7500 Hz. Multiple cells were recorded by each electrode and cells sorting was carried out using MATLAB software (MathWorks Inc., Natick, MA, USA). Data analysis was conducted by Neuroexplored4 (Plexon Inc.). According to previous study,^[^ ^6a^ ^]^ in vivo bursting was defined as clusters of spikes with an initial inter‐spike interval no greater than 20 ms and a final inter‐spike interval no greater than 100 ms. The minimum intra‐burst interval was established at 100 ms, and a burst was defined as having a minimum of 2 spikes.

### Statistical Analysis

All statistics were analyzed using GraphPad Prism 9 software. Researchers who were blinded to the experimental circumstances conducted the data analysis. The Figures and Figure legends contain statistical information, such as definitions and precise values of n (for example, the number of mice or neurons), *p* values, and the types of the statistical tests. In order to compare the data from the two groups, the homoscedasticity and normality of the distributions of data were determined before assigning specific statistical tests. For data following a normal distribution, paired and unpaired Student's *t*‐tests were utilized. For data that did not follow a normal distribution, the Mann‐Whitney U test and the Wilcoxon signed‐rank test were applied. For the analysis of data from three or more groups, one‐way ANOVA and two‐way ANOVA was used. The percentage of burst‐firing neurons was calculated using Chi‐square test. For all figures, the horizontal lines in the dot plots represent the mean. Statistical significance was set at *p* < 0.05.

## Conflict of Interest

The authors declare no conflict of interest.

## Author Contributions

C.R., S.L., L.H., Q.T. designed experiments and wrote the manuscript. X.L. performed surgery, in vivo optetrode recordings, and microscopy. X.L., H.L., R.M., and X.T. performed behavioral experiments. S.L., X.H., and J.W. performed physiological recordings. J.W. assisted with behavioral experiments. H.L., R.M., and X.T. assisted with the analysis of virus tracing data. C.R., S.L., L.H., X.L., and K.S. analyzed the data.

## Supporting information

Supporting Information

## Data Availability

The data that support the findings of this study are available from the corresponding author upon reasonable request.
