# Supplementary material for: Burst firing in Output‐Defined Parallel Habenula Circuit Underlies the Antidepressant Effects of Bright Light Treatment
Source: Adv Sci (Weinh). 2024 Jun 11;11(30):2401059. doi: 10.1002/advs.202401059 (PMC11321664; doi:10.1002/advs.202401059)
Supplement: Supplementary file 1 — Supporting Information [file ADVS-11-2401059-s001.pdf]

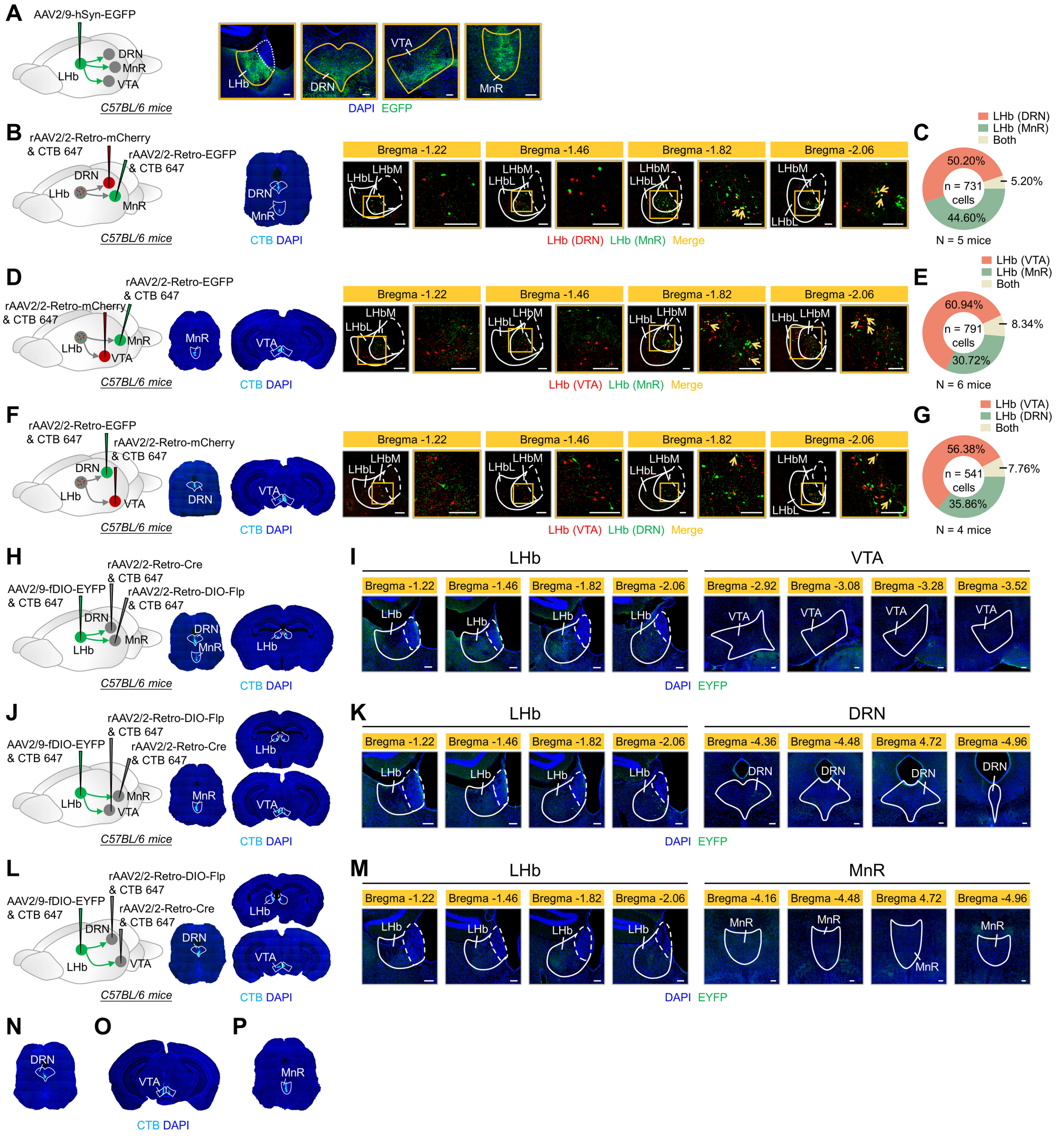

Figure S1. LHb$^{\rightarrow DRN}$, LHb$^{\rightarrow VTA}$, and LHb$^{\rightarrow MnR}$ neurons are distinct subpopulations. A) Scheme for specific infection of LHb neurons with EGFP and representative images of axons in DRN, VTA, and MnR from LHb neurons. Scale bar: 100 μm. B) Retrograde labelling of LHb$^{\rightarrow MnR}$ and LHb$^{\rightarrow DRN}$ neurons by injecting rAAV2/2-Retro-EGFP into the MnR and rAAV2/2-Retro-mCherry into the DRN, respectively. CTB-647 for injection site visualization (left). Confocal micrographs of LHb$^{\rightarrow MnR}$ with EGFP, LHb$^{\rightarrow DRN}$ neurons with mCherry and co-labeled LHb neurons from the anterior to posterior poles and the lateral and medial parts of the LHb (right). Scale bar: 100 μm. C) Proportion of co-labeled LHb$^{\rightarrow MnR}$ and LHb$^{\rightarrow DRN}$ neurons (N = 5 mice). D) Retrograde labelling of LHb$^{\rightarrow MnR}$ and LHb$^{\rightarrow VTA}$ neurons by injecting rAAV2/2-Retro-EGFP into the MnR and rAAV2/2-Retro-mCherry into the VTA, respectively. CTB-647 for injection site visualization (left). Confocal

micrographs of $LHb^{\rightarrow MnR}$ with EGFP, $LHb^{\rightarrow VTA}$ neurons with mCherry and co-labeled LHb neurons from the anterior to posterior poles and the lateral and medial parts of the LHb (right). Scale bar: 100 μm. E) Proportion of co-labeled $LHb^{\rightarrow MnR}$ and $LHb^{\rightarrow VTA}$ neurons (N = 6 mice). F) Retrograde labelling of $LHb^{\rightarrow DRN}$ and $LHb^{\rightarrow VTA}$ neurons by injecting rAAV2/2-Retro-EGFP into the DRN and rAAV2/2-Retro-mCherry into the VTA, respectively. CTB-647 for injection site visualization (left). Confocal micrographs of $LHb^{\rightarrow DRN}$ with EGFP, $LHb^{\rightarrow VTA}$ neurons with mCherry and co-labeled LHb neurons from the anterior to posterior poles and the lateral and medial parts of the LHb (right). Scale bar: 100 μm. G) Proportion of co-labeled $LHb^{\rightarrow DRN}$ and $LHb^{\rightarrow VTA}$ neurons (N = 4 mice). H) Scheme for retrograde labelling of LHb neurons by injecting rAAV2/2-Retro-Cre into the DRN, rAAV2/2-Retro-DIO-Flp into MnR and AAV2/9-fDIO-EYFP into LHb. CTB-647 for injection site visualization. I) Confocal micrographs of the LHb neurons from the anterior to posterior poles targeting both DRN and MnR, and the axon in VTA from anterior to posterior. Scale bar: 100 μm. J) Scheme for retrograde labelling of LHb neurons by injecting rAAV2/2-Retro-Cre into the MnR, rAAV2/2-Retro-DIO-Flp into VTA and AAV2/9-fDIO-EYFP into LHb. CTB-647 for injection site visualization. K) Confocal micrographs of the LHb neurons from the anterior to posterior poles targeting both MnR and VTA, and the axon in DRN from anterior to posterior. Scale bar: 100 μm. L) Scheme for retrograde labelling of LHb neurons by injecting rAAV2/2-Retro-Cre into the VTA, rAAV2/2-Retro-DIO-Flp into DRN and AAV2/9-fDIO-EYFP into LHb. CTB-647 for injection site visualization. M) Confocal micrographs of the LHb neurons from the anterior to posterior poles targeting both VTA and DRN, and the axon in MnR from anterior to posterior. Scale bar: 100 μm. N) Location of the injection site of rAAV2/2-Retro-Cre in DRN was visualized by CTB-647. O) Location of the injection site of rAAV2/2-Retro-Cre in VTA was visualized by CTB-647. P) Location of the injection site of rAAV2/2-Retro-Cre in MnR was visualized by CTB-647.

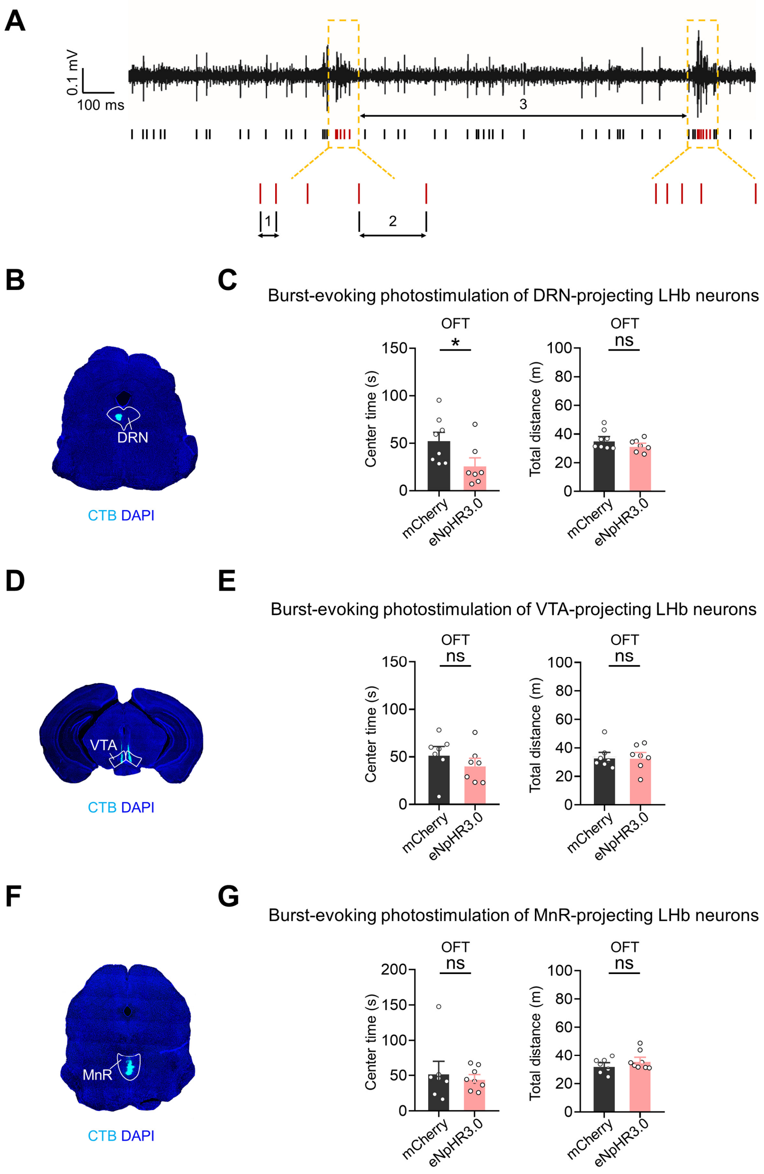

Figure S2. eNpHR3.0-induced rebound bursting in LHb$^{\rightarrow DRN}$ increases anxiety-like behavior. A) An example trace of burst events of LHb neurons in an *in vivo* recording. Bursts (red sticks) are identified utilizing the inter-spike interval method (refer to Methods section): 1. Burst initiation; 2. Burst termination; 3. Inter-burst interval. B) Location of the injection site of rAAV2/2-Retro-Cre in the DRN was visualized by CTB-647. C) Anxiety-like behavior (left) and locomotor activity (right) of mice in different experimental groups (mCherry: N =8 mice; eNpHR3.0: N = 7 mice), data presents Mean ± SEM; Two tail unpaired t-test, *, $P<0.05$; ns, no significant difference. All mice received DRN injection of rAAV2/2-Retro-Cre and CTB-647. mCherry, mice that received LHb injection of AAV2/9-DIO-mCherry; eNpHR3.0, mice that received LHb injection of AAV2/9-DIO-eNpHR3.0-mCherry. D) Location of the injection site of rAAV2/2-Retro-Cre in the VTA was

visualized by CTB-647. E) Anxiety-like behavior (left) and locomotor activity (right) of mice in different experimental groups (mCherry: N =7 mice; eNpHR3.0: N = 7 mice), data presents Mean ± SEM; Two tail unpaired t-test, ns, no significant difference. All mice received VTA injection of rAAV2/2-Retro-Cre and CTB-647. mCherry, mice that received LHb injection of AAV2/9-DIO-mCherry; eNpHR3.0, mice that received LHb injection of AAV2/9-DIO-eNpHR3.0-mCherry. F) Location of the injection site of rAAV2/2-Retro-Cre in the MnR was visualized by CTB-647. G) Anxiety-like behavior (left) and locomotor activity (right) of mice in different experimental groups (mCherry: N =7 mice; eNpHR3.0: N = 8 mice), data presents Mean ± SEM; Mann-Whitney U test, ns, no significant difference. All mice received MnR injection of rAAV2/2-Retro-Cre and CTB-647. mCherry, mice that received LHb injection of AAV2/9-DIO-mCherry; eNpHR3.0, mice that received LHb injection of AAV2/9-DIO-eNpHR3.0-mCherry.

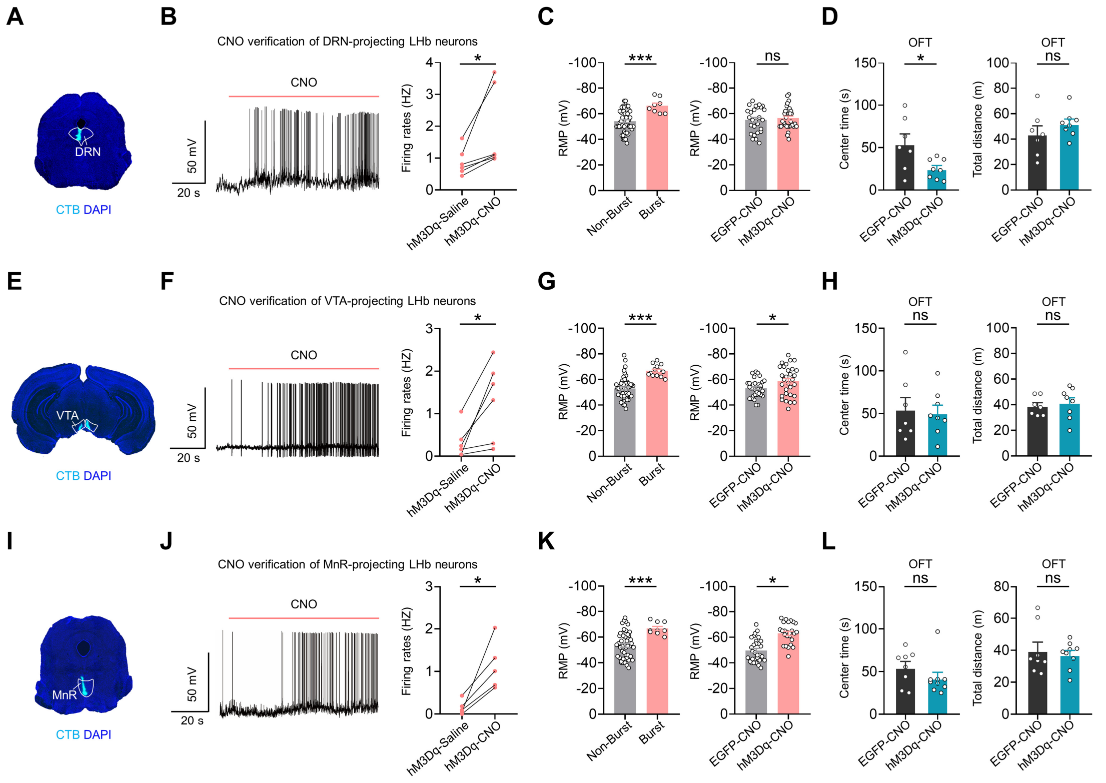

Figure S3. Verification of chemogenetic manipulation and the effect on both RMP and anxiety-like behavior when long-term activation of the $LHb^{\rightarrow DRN}$, $LHb^{\rightarrow VTA}$, and $LHb^{\rightarrow MnR}$ neurons. A-D) Location of the injection site of rAAV2/2-Retro-Cre in the DRN was visualized by CTB-647 (A). $LHb^{\rightarrow DRN}$ neurons expressing hM3Dq were activated by bath application of CNO (10 μM) (n = 6 cells, N = 3 mice), data presents Wilcoxon matched-pairs signed rank test, *, P<0.05 (B); RMP of the Non-bursting $LHb^{\rightarrow DRN}$ neurons (n = 50 cells, N = 9 mice) and bursting $LHb^{\rightarrow DRN}$ neurons (n = 8 cells, N = 9 mice) (left), and RMP of the $LHb^{\rightarrow DRN}$ neurons in EGFP-CNO (n = 27 cells, N = 5 mice) and hM3Dq-CNO (n = 31 cells, N = 4 mice) groups (right), data presents Mean ± SEM; Two tail unpaired t-test, ***, P<0.001; ns, no significant difference. (C); Anxiety-like behavior (left) and locomotor activity (right) of mice in different experimental groups (EGFP-CNO: N = 7 mice; hM3Dq-CNO: N = 8 mice), data presents Mean ± SEM; Two tail unpaired t-test, *, P<0.05; ns, no significant difference (D). All mice received DRN injection of rAAV2/2-Retro-Cre and CTB-647. EGFP-CNO, mice that received LHb injection of AAV2/9-DIO-EGFP and i.p. injection of CNO (1 mg/kg); hM3Dq-CNO, mice that received LHb injection of AAV2/9-DIO-hM3Dq-EGFP and i.p. injection of CNO (1 mg/kg). E-H) Location of the injection site of rAAV2/2-Retro-Cre in the VTA was visualized by CTB-647 (E). $LHb^{\rightarrow VTA}$ neurons expressing hM3Dq were activated by bath application of CNO (10 μM) (n = 6 cells, N = 3 mice), data presents Two tail paired t-test, *, P<0.05

(F); RMP of the Non-bursting LHb$^{\rightarrow VTA}$ neurons (n = 51 cells, N = 8 mice) and bursting LHb$^{\rightarrow VTA}$ neurons (n = 13 cells, N = 8 mice) (left), and RMP of the LHb$^{\rightarrow VTA}$ neurons in EGFP-CNO (n = 34 cells, N = 4 mice) and hM3Dq-CNO (n = 30 cells, N = 4 mice) groups (right), data presents Mean ± SEM; Two tail unpaired t-test, *, P<0.05; ***, P<0.001 (G); Anxiety-like behavior (left) and locomotor activity (right) of mice in different experimental groups (EGFP-CNO: N = 7 mice; hM3Dq-CNO: N = 8 mice), data presents Mean ± SEM; Two tail unpaired t-test, ns, no significant difference (H). All mice received VTA injection of rAAV2/2-Retro-Cre and CTB-647. EGFP-CNO, mice that received LHb injection of AAV2/9-DIO-EGFP and i.p. injection of CNO (1 mg/kg); hM3Dq-CNO, mice that received LHb injection of AAV2/9-DIO-hM3Dq-EGFP and i.p. injection of CNO (1 mg/kg). I-L) Location of the injection site of rAAV2/2-Retro-Cre in the MnR was visualized by CTB-647 (I). LHb$^{\rightarrow MnR}$ neurons expressing hM3Dq were activated by bath application of CNO (10 μM) (n = 5 cells, N = 3 mice), Data presents Two tail paired t-test, *, P<0.05 (J); RMP of the Non-bursting LHb$^{\rightarrow MnR}$ neurons (n = 46 cells, N = 9 mice) and bursting LHb$^{\rightarrow MnR}$ neurons (n = 9 cells, N = 9 mice) (left), and RMP of the LHb$^{\rightarrow MnR}$ in EGFP-CNO (n = 30 cells, N = 5 mice) and hM3Dq-CNO (n = 25 cells, N = 4 mice) groups (right), data presents Mean ± SEM; Two tail unpaired t-test, *, P<0.05; ***, P<0.001 (K); Anxiety-like behavior (left) and locomotor activity (right) of mice in different experimental groups (EGFP-CNO: N = 8 mice; hM3Dq-CNO: N = 9 mice), data presents Mean ± SEM; Mann-Whitney U test, ns, no significant difference (L). All mice received MnR injection of rAAV2/2-Retro-Cre and CTB-647. EGFP-CNO, mice that received LHb injection of AAV2/9-DIO-EGFP and i.p. injection of CNO (1 mg/kg); hM3Dq-CNO, mice that received LHb injection of AAV2/9-DIO-hM3Dq-EGFP and i.p. injection of CNO (1 mg/kg).

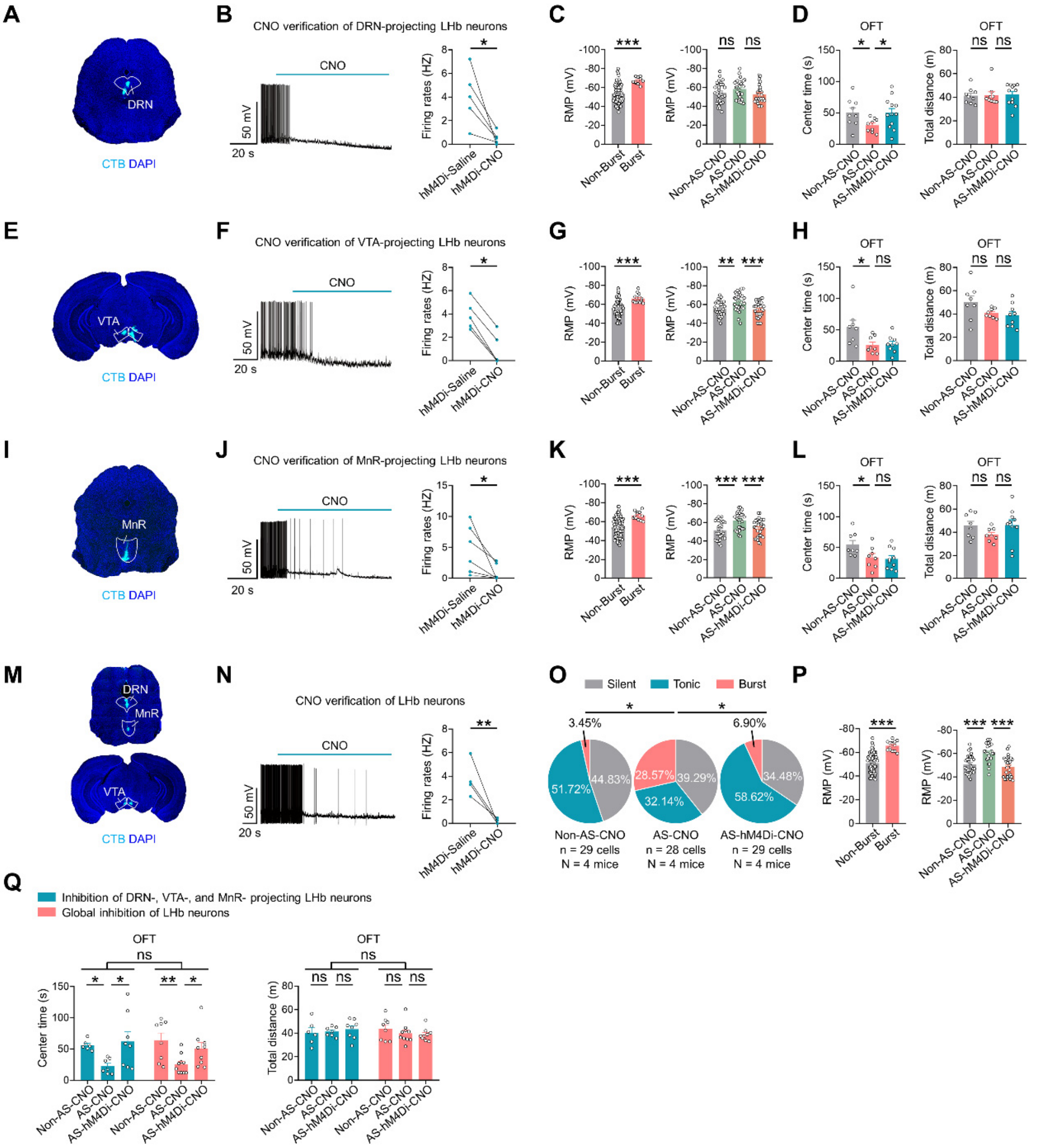

Figure S4. Verification of chemogenetic manipulation and the effect on both RMP and anxiety-like behaviors when long-term inhibition of the LHb$^{\rightarrow DRN}$, LHb$^{\rightarrow VTA}$, and LHb$^{\rightarrow MnR}$ neurons and long-term global inhibition of LHb neurons. A-D) Location of the injection site of rAAV2/2-Retro-Cre in the DRN was visualized by CTB-647 (A); LHb$^{\rightarrow DRN}$ neurons expressing hM4Di were inhibited by bath application of CNO (10 μM) (n = 5 cells, N = 3 mice), data presents Two tail paired t-test, *, P<0.05 (B); RMP of the Non-bursting LHb$^{\rightarrow DRN}$ neurons (n = 80 cells, N = 13 mice) and bursting LHb$^{\rightarrow DRN}$ neurons (n = 11 cells, N = 13 mice) (left), and RMP of the LHb$^{\rightarrow DRN}$ neurons in Non-AS-CNO (n = 30 cells, N = 4 mice), AS-CNO (n = 34 cells, N = 5 mice), and AS-hM4Di-CNO (n = 27 cells, N = 4 mice) groups (right), data presents Mean ± SEM; Two tail unpaired t-test and One-way ANOVA with post hoc LSD test, ***, *P*<0.001; ns, no significant difference (C); Anxiety-like

behavior (left) and locomotor activity (right) of mice in different experimental groups Non-AS-CNO: N = 9 mice; AS-CNO: N = 9 mice; AS-hM4Di-CNO: N = 12 mice), data presents Mean ± SEM; One-way ANOVA with post hoc LSD test, *, *P*<0.05; ns, no significant difference (D). All mice received DRN injection of rAAV2/2-Retro-Cre and CTB-647. Non-AS-CNO, mice that received LHb injection of AAV2/9-DIO-EYFP, i.p. injection of CNO (1 mg/kg), and no exposure to AS; AS-CNO, mice that received LHb injection of AAV2/9-DIO-EYFP, i.p. injection of CNO (1 mg/kg), and exposure to AS; AS-hM4Di-CNO, mice that received LHb injection of AAV2/9-DIO-hM4Di-EYFP, i.p. injection of CNO (1 mg/kg), and exposure to AS. E-H) Location of the injection site of rAAV2/2-Retro-Cre in the VTA was visualized by CTB-647 (E); $\text{LHb}^{\rightarrow \text{VTA}}$ neurons expressing hM4Di were inhibited by bath application of CNO (10 μM) (n = 5 cells, N = 3 mice), data presents Two tail paired t-test, *, P<0.05 (F); RMP of the Non-bursting $\text{LHb}^{\rightarrow \text{VTA}}$ neurons (n = 75 cells, N = 13 mice) and bursting $\text{LHb}^{\rightarrow \text{VTA}}$ neurons (n = 15 cells, N = 13 mice) (left), and RMP of the $\text{LHb}^{\rightarrow \text{VTA}}$ neurons in Non-AS-CNO (n = 32 cells, N = 4 mice), AS-CNO (n = 29 cells, N = 5 mice), and AS-hM4Di-CNO (n = 29 cells, N = 4 mice) groups (right), data presents Mean ± SEM; Two tail unpaired t-test and One-way ANOVA with post hoc LSD test, **, *P*<0.01;***, *P*<0.001 (G); Anxiety-like behavior (left) and locomotor activity (right) of mice in different experimental groups Non-AS-CNO: N = 8 mice; AS-CNO: N = 8 mice; AS-hM4Di-CNO: N = 9 mice), data presents Mean ± SEM; One-way ANOVA with post hoc LSD test, *, P<0.05; ns, no significant difference (H). All mice received VTA injection of rAAV2/2-Retro-Cre and CTB-647. Non-AS-CNO, mice that received LHb injection of AAV2/9-DIO-EYFP, i.p. injection of CNO (1 mg/kg), and no exposure to AS; AS-CNO, mice that received LHb injection of AAV2/9-DIO-EYFP, i.p. injection of CNO (1 mg/kg), and exposure to AS; AS-hM4Di-CNO, mice that received LHb injection of AAV2/9-DIO-hM4Di-EYFP, i.p. injection of CNO (1 mg/kg), and exposure to AS. I-L) Location of the injection site of rAAV2/2-Retro-Cre in the MnR was visualized by CTB-647 (I); $\text{LHb}^{\rightarrow \text{MnR}}$ neurons expressing hM4Di were inhibited by bath application of CNO (10 μM) (n = 6 cells, N = 4 mice), data presents Two tail paired t-test, *, P<0.05 (J); RMP of the Non-bursting $\text{LHb}^{\rightarrow \text{MnR}}$ neurons (n = 82 cells, N = 12 mice) and bursting $\text{LHb}^{\rightarrow \text{MnR}}$ neurons (n = 13 cells, N = 12 mice) (left), and RMP of the $\text{LHb}^{\rightarrow \text{MnR}}$ neurons in Non-AS-CNO (n = 30 cells, N = 4 mice), AS-CNO (n = 31 cells, N = 4 mice), and AS-hM4Di-CNO (n = 34 cells, N = 4 mice) groups, data presents Mean ± SEM; Two tail unpaired t-test and One-way ANOVA with post hoc LSD test, ***,

P<0.001 (right) (K); Anxiety-like behavior (left) and locomotor activity (right) of mice in different experimental groups Non-AS-CNO: N = 8 mice; AS-CNO: N = 8 mice; AS-hM4Di-CNO: N = 10 mice), data presents Mean ± SEM; One-way ANOVA with post hoc LSD test, *, P<0.05; ns, no significant difference (L). All mice received MnR injection of rAAV2/2-Retro-Cre and CTB-647. Non-AS-CNO, mice that received LHb injection of AAV2/9-DIO-EYFP, i.p. injection of CNO (1 mg/kg), and no exposure to AS; AS-CNO, mice that received LHb injection of AAV2/9-DIO-EYFP, i.p. injection of CNO (1 mg/kg), and exposure to AS; AS-hM4Di-CNO, mice that received LHb injection of AAV2/9-DIO-hM4Di-EYFP, i.p. injection of CNO (1 mg/kg), and exposure to AS. M-Q) Location of the injection site of rAAV2/2-Retro-Cre in the DRN, VTA, and MnR was visualized by CTB-647 (M). LHb neurons expressing hM4Di were inhibited by bath application of CNO (10 μM) (n = 4 cells, N = 2 mice), data presents Two tail paired t-test, **, P<0.01 (N); Pie charts indicate percentages of the three types of LHb neurons in Non-AS-CNO (n = 29 cells, N = 4 mice), AS-CNO (n = 28 cells, N = 4 mice), and AS-hM4Di-CNO (n = 29 cells, N = 4 mice) groups, data presents chi-square test, *, P<0.05 (O). RMP of the Non-bursting LHb neurons (n = 72 cells, N = 12 mice) and bursting LHb neurons (n = 14 cells, N = 12 mice) (left), and RMP of the LHb neurons in Non-AS-CNO (n = 29 cells, N = 4 mice), AS-CNO (n = 28 cells, N = 4 mice), and AS-hM4Di-CNO (n = 29 cells, N = 4 mice) groups, data presents Mean ± SEM; Two tail unpaired t-test and One-way ANOVA with post hoc LSD test, ***, *P*<0.001 (P). Anxiety-like behavior (left) and locomotor activity (right) of mice in different experimental groups (blue label: Non-AS-CNO: N = 6 mice; AS-CNO: N = 7 mice; AS-hM4Di-CNO: N = 8 mice) (red label: Non-AS-CNO: N = 8 mice; AS-CNO: N = 10 mice; AS-hM4Di-CNO: N = 9 mice), data presents Mean ± SEM; One-way ANOVA with post hoc LSD test and Two-way ANOVA with Sidak's multiple-comparisons test, *, P<0.05; **, P<0.01; ns, no significant difference (Q); For simultaneous inhibition of DRN-, VTA-, and MnR-projecting LHb neurons, all mice received DRN, VTA, and MnR injection of rAAV2/2-Retro-Cre and CTB-647, and LHb injection of AAV2/9-DIO-EYFP or AAV2/9-DIO-hM4Di-EYFP. For global inhibition of LHb neurons, all mice received LHb injection of AAV2/9-hSyn-EGFP or AAV2/9-hSyn-hM4Di-EGFP. Non-AS-CNO, mice with EYFP or EGFP expressed in LHb, received i.p. injection of CNO (1 mg/kg), and no exposure to AS; AS-CNO, mice with EYFP or EGFP expressed in LHb, received i.p. injection of CNO (1 mg/kg), and exposure to AS; AS-hM4Di-CNO, mice with hM4Di expressed in LHb, received i.p. injection of CNO (1 mg/kg), and exposure to AS.

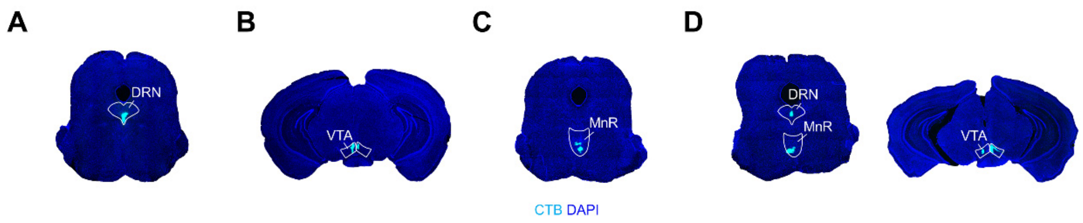

Figure S5. Location of the injection site of rAAV2/2-Retro-Cre in the DRN, VTA and MnR. A) Location of the injection site of rAAV2/2-Retro-Cre in the DRN was visualized by CTB-647. B) Location of the injection site of rAAV2/2-Retro-Cre in the VTA was visualized by CTB-647. C) Location of the injection site of rAAV2/2-Retro-Cre in the MnR was visualized by CTB-647. D) Location of the injection site of rAAV2/2-Retro-Cre in the DRN, VTA and MnR was visualized by CTB-647.
